# Programmable Elastic Wave Control Via Mechanical‐Acoustic Interaction in Bistable Metamaterials

**DOI:** 10.1002/advs.76005

**Published:** 2026-06-12

**Authors:** Yuanyuan Li, Yonghua Yu, Chuanqing Chen, Rui Xu, Yulong He, Xin Li, Ming‐Hui Lu, Yan‐Feng Chen

**Affiliations:** ^1^ National Laboratory of Solid State Microstructures Nanjing University Nanjing China; ^2^ College of Engineering and Applied Sciences Nanjing University Nanjing China; ^3^ School of Mechanical Engineering Nanjing University of Science and Technology Nanjing China; ^4^ Jiangsu Physical Science Research Center Nanjing China

**Keywords:** acoustics, attenuation, bistability, mechanics, metamaterial, modular design, scalability, vibration, wave propagation

## Abstract

Conventional elastic wave metamaterials are typically constrained by fixed structural configurations, which limits their ability to achieve reconfigurable and application‐specific wave manipulation. To address this challenge, this work propose a mechanical–acoustic interaction (MAI) paradigm for mechanically programmable elastic wave control, in which acoustic functionalities are reconfigured through reversible switching between bistable mechanical states. The proposed acoustic dome metamaterial (ADM) consists of modular bistable units, where the peak and valley configurations serve as two mechanically programmable states without relying on electrical, magnetic, or thermal stimuli. By spatially encoding these bistable states, the band structure and transmission characteristics of the metamaterial can be reconfigured, enabling programmable control of elastic wave propagation, attenuation, and energy localization. Numerical and experimental results demonstrate low‐frequency vibration suppression, reconfigurable waveguiding, and defect‐state‐enabled energy localization governed by mechanically encoded state patterns. Moreover, a one‐press programming strategy is introduced to improve programming efficiency and reproducibility at the system level. These findings establish MAI as a physically intuitive and scalable mechanism for elastic wave programming, offering new opportunities for reconfigurable acoustic metamaterials and intelligent acoustic devices.

## Graphical abstract

A mechanically programmable metamaterial based on mechanical‐acoustic interaction enables reconfigurable elastic wave control through bistable state switching. Spatial encoding of peak and valley states tunes band structures and transmission, achieving low‐frequency vibration suppression, waveguiding, and energy localization. A one‐press strategy enhances programming efficiency, establishing a scalable paradigm for reconfigurable acoustic devices.

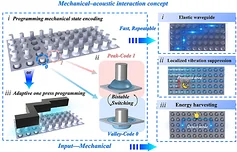

## 1 Introduction

The effective control of elastic waves plays a crucial role in a broad range of applications, including vibration suppression [1, 2], acoustic information processing [3, 4], energy harvesting [5, 6, 7], and structural health monitoring [8, 9]. The emergence of elastic wave metamaterials has offered new opportunities for manipulating wave propagation beyond the limits of conventional materials [10, 11, 12, 13]. Through deliberately designed unit architectures, these metamaterials can exhibit extraordinary functionalities such as bandgap formation [11, 14, 15], energy localization [16, 17], and waveguiding [18, 19, 20], thereby providing powerful routes for controlling vibration and elastic energy transport.

Despite these advances, most elastic wave metamaterials remain static once fabricated, and their dynamic responses cannot be flexibly adjusted after manufacture. To overcome this limitation, considerable efforts have been devoted to tunable and reconfigurable metamaterials. One important class relies on external stimuli, including electric fields [21], magnetic fields [22], thermal loading [23], and optical excitation [24], to regulate wave responses. For instance, local resonance bandgap control has been effectively achieved in particle‐aligned magnetorheological acoustic metamaterials [25]. These approaches provide effective and often rapid tunability. However, they usually depend on relatively complex driving systems, external field generation, or sustained energy input, which increases system complexity and may limit their practicality in engineering scenarios requiring structural simplicity, long‐term stability, or low power consumption.

Along with active modulation, structural reconfiguration [26, 27], often inspired by shape‐morphing metamaterials such as origami and kirigami, provides an alternative route for elastic‐wave manipulation [28, 29]. By directly altering the lattice geometry, these systems can achieve significant functional shifts without relying on complex external fields. Recent review articles on structural reconfiguration have further summarized the underlying design principles of such systems [30, 31, 32]. However, many existing structurally reconfigurable metamaterials focus primarily on global shape adaptation, static property switching, or overall tunability, while unit‐level independent addressing and spatially encoded multifunctional wave control remain relatively underexplored.

More recently, the development of programmable metamaterials [33, 34] has extended this field beyond tunability alone, toward logic, information processing, wave‐based computing, and mechanical intelligence [35, 36, 37]. These studies show that metamaterials can serve not only as wave‐control media but also as platforms for encoding functional rules through structured state distributions. This trend is particularly relevant to acoustic and elastic systems, where local resonance and spatial state arrangement can be harnessed to realize increasingly sophisticated functionalities. However, not all practical wave‐control scenarios require tunable or programmable metamaterials with continuous real‐time adaptability. For applications where the desired wave functionality is predefined or only infrequently updated, such as preset vibration isolation, reconfigurable waveguiding, and energy localization, mechanically programmable platforms that enable stable state locking without continuous energy input are also highly valuable.

**FIGURE 1:**
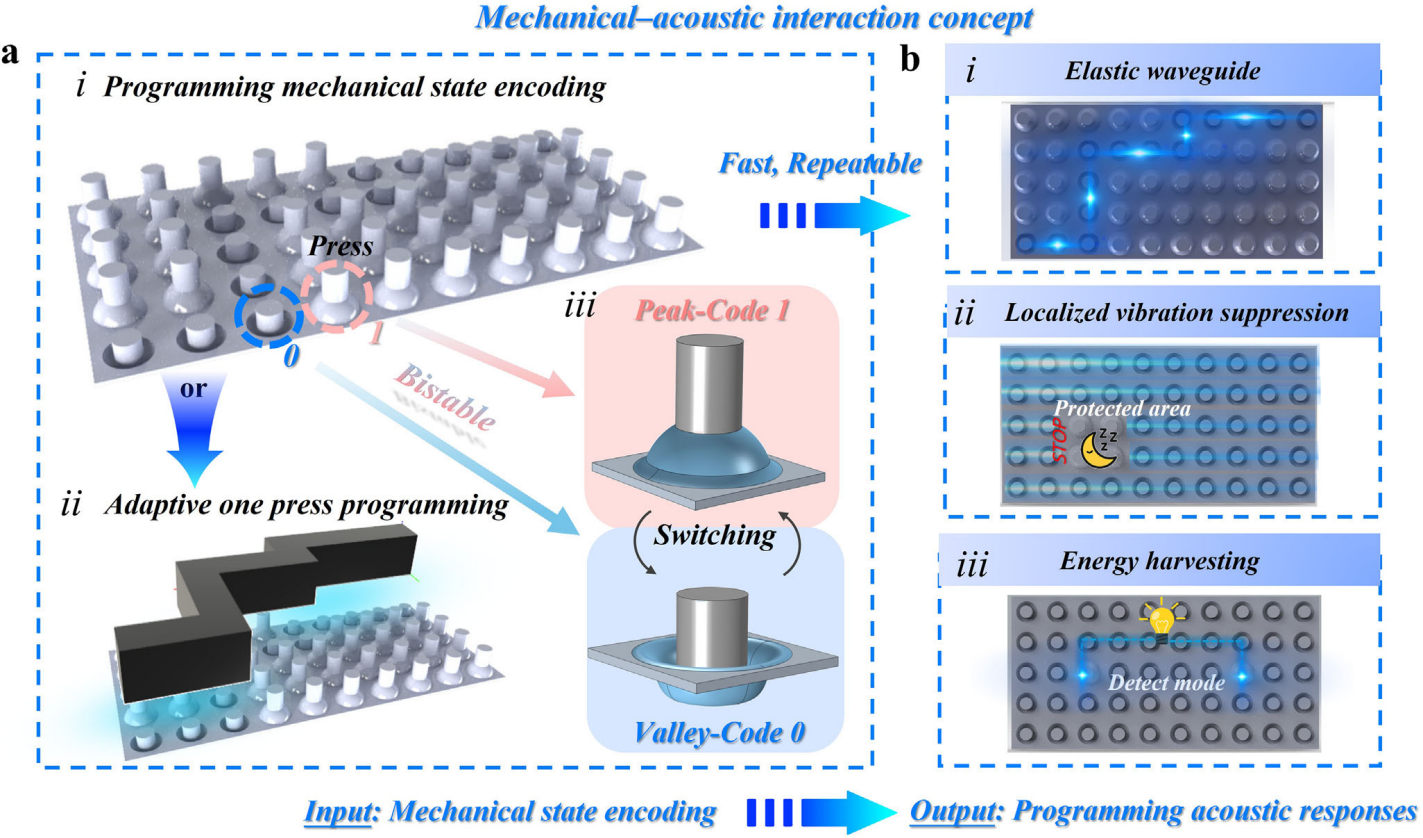
Schematic illustration of the MAI concept in the ADM. (a) Input: Mechanical state encoding of bistable unit mechanical states (0/1), achieved either by (i) sequential single‐unit pressing or by (ii) adaptive one‐press programming. (b) Output: Programmable acoustic responses and different acoustic functionalities, including (i) elastic waveguiding, (ii) localized vibration suppression, and (iii) energy harvesting.

Motivated by these considerations, this work introduces a mechanical–acoustic interaction (MAI) mechanism based on bistable units, providing a new route to programmable and reconfigurable elastic wave control, as illustrated in Figure 1. Compared with existing stimuli‐responsive or globally reconfigurable metamaterials, the proposed paradigm offers several distinctive advantages. First, it enables programmable elastic‐wave manipulation without relying on continuous electrical, magnetic, or thermal fields, which is favorable for simplified system design, reduced energy dependence, and stable state retention. Second, the bistable architecture supports unit‐level independent programming, allowing multiple wave functionalities to be encoded through spatially distributed mechanical states. However, unlike continuously adaptive systems, the present framework is not intended for autonomous real‐time reconfiguration, but is instead better suited to scenarios requiring stable, mechanically written state distributions. It should be noted that the focus of the present work is not on the design of specific programming actuation methods, but rather on the mechanistic foundation and functional potential of bistable shell structures as a physically programmable platform for elastic‐wave control. Based on this structural platform, more efficient actuation and integration strategies can be further developed in the future to meet the needs of different application scenarios.

Conceptually, this MAI mechanism can be understood as a physically implemented input–output interaction, in which externally applied mechanical inputs are translated into reconfigurable wave functionalities, without requiring electronic interfaces or digital control systems (see Figure S1). On this basis, an acoustic dome metamaterial (ADM) is proposed, in which the peak and valley configurations of bistable units are encoded as binary mechanical states. By spatially encoding these mechanically writable states, the local interaction landscape of the metamaterial can be reconfigured, enabling programmable manipulation of elastic wave propagation, attenuation, and energy localization. Experimental and numerical results further demonstrate reconfigurable waveguiding, localized vibration suppression, and programmable defect‐state energy harvesting. In addition, a one‐press programming strategy enabled by structural shape adaptability is introduced as an efficient programming interface to improve writing efficiency and reproducibility at the system level. Overall, this work establishes MAI as a physically intuitive, reversible, and efficient mechanism for mechanically programmable elastic‐wave control, thereby offering a new design route toward robust and multifunctional reconfigurable acoustic metamaterials.

## 2 Results and Discussion

### 2.1 MAI–Enabled ADM Design and Bandgap Mechanism

MAI in this work refers to a state‐dependent coupling between mechanical configurations and elastic wave responses, which enables reversible and programmable modulation of wave propagation through mechanical state switching (Figure 1). By applying external mechanical stimuli, bistable units can be selectively switched between distinct stable configurations (Figure 1a(iii)), thereby changing the local state distribution within the metamaterial and, consequently, its acoustic response (Figure 1b).

**FIGURE 2:**
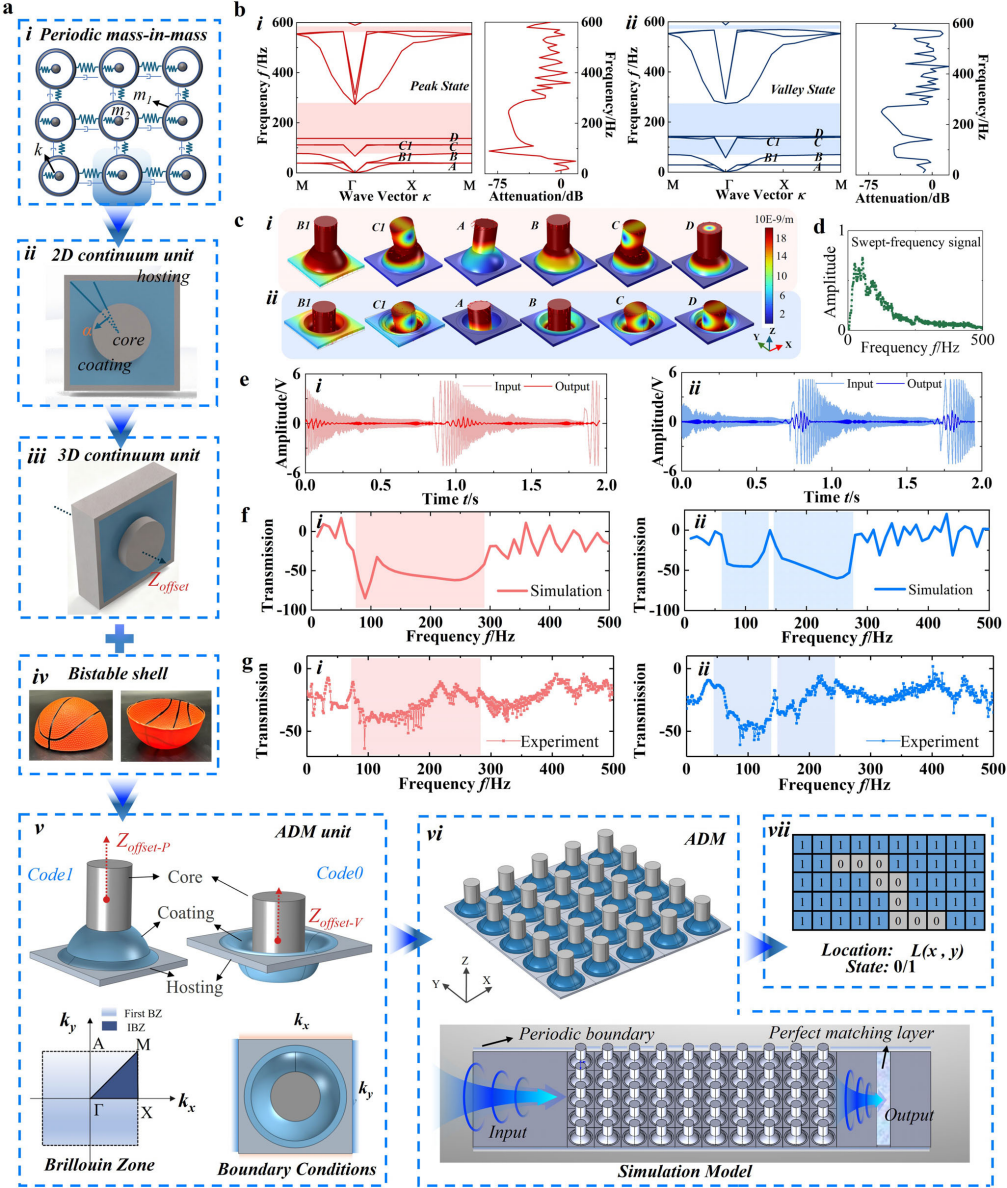
Design concept and acoustic responses of the ADM. (a) Schematic illustration of the design evolution of the ADM. (i) classical periodic mass‐in‐mass lattice; (ii) a 2D continuous LRAM unit corresponding to a single unit cell extracted from the periodic mass‐in‐mass lattice. When extended to 3D, (iii) the vertical position of the resonant core introduces a geometric parameter, denoted as the centroid offset $Z_{\text{offset}}$, which plays a critical role in tailoring the band structure characteristics. By combining the effect of $Z_{\text{offset}}$ with (iv) the bistable mechanical behavior of the spherical dome, (v) an ADM unit with bistable characteristics is designed. The unit cell Brillouin zone and Bloch boundary conditions are also displayed. Periodic arrangement of such units results in (vi) the ADM, whose spatial bistable configurations enable (vii) programmable elastic wave responses. (b) Dispersion relations of the ADM in the (i) peak state and (ii) valley state, respectively, where the shaded regions denote the acoustic bandgaps. The frequency ranges of strong elastic wave attenuation, characterized by the transmission coefficients, exhibit a clear correspondence with the predicted bandgaps. (c) Representative vibration modes and displacement vector fields of the ADM unit cell in the peak state and the valley state, each corresponding one‐to‐one to the labeled points *B1*, *C1*, *A*, *B*, *C* and *D* in the dispersion curves. (d–g) ADM based on a finite periodic lattice consisting of 10 × 5 unit cells: i) peak state and ii)the valley state. (d) Frequency domain of the input signal ($f_{e}$ = 10‐500 Hz, $V_{p}=3\;V$). (e) Time‐domain input and output vibration signals. (f) Simulated transmission coefficient curves for comparison. (g) Experimentally measured transmission coefficient curves, showing good agreement with the numerical predictions.

The physical origin of this interaction can be understood within the established theoretical framework of locally resonant acoustic metamaterials (LRAMs). As illustrated in Figure 2a(i–iii), the band structure sensitivity to the core centroid offset in 3D continuous LRAMs can be explained by the mass‐in‐mass model and 2D design principles (see Section S1). This theoretical consideration indicates that geometric asymmetry in the resonant unit plays a critical role in tailoring local resonance characteristics and wave dispersion.

In the field of mechanical metamaterials, spherical dome shaped shells constitute a prototypical 3D bistable structure [38]. As illustrated in Figure 2a(iv), such shells exhibit two mechanically stable equilibria, enabling reversible state switching upon mechanical loading (see Movies S1 and S2). Motivated by the combined effects of centroid‐offset‐induced acoustic modulation and reversible bistability, a bistable ADM unit is developed. As shown in Figures 2a(v,vi), each ADM unit consists of three primary components: a lead mass block (core), a silicone dome shell (coating), and a polylactic acid matrix (hosting). Its bistable mechanical characteristics are critical for achieving programmability and reconfigurability in the metamaterial. Specifically, the two bistable states serve as switchable configurations, while the negative stiffness enhances the sensitivity of local transitions, enabling dynamic control of elastic wave propagation at the unit‐cell level.(see Sections S1 and S2). Under mechanical compression, the unit can reversibly transition between a convex “peak” state and a concave “valley” state, which are encoded as binary states 1 and 0, respectively. (see Sections S3 and S6) The periodic arrangement of ADM units maps spatial coordinates L(x,y) directly to programmed mechanical states. This allows for the spatial manipulation of elastic wave responses, as shown in Figure 2a(vii).

In this context, the proposed MAI provides a physical mechanism that links mechanical actuation to acoustic functionality, forming the basis for programmable elastic wave manipulation in the metamaterial. To clarify how the programmed bistable states produce different acoustic responses, the formation and evolution of bandgaps in the ADM were analyzed. Within the established framework of continuous LRAMs, the band structure is primarily governed by the mass ratio of the resonant unit and the effective stiffness of the surrounding layer. The acoustic bandgap originates from out‐of‐phase motion induced by local resonance, which generates a reactive force field suppressing wave propagation, with the upper and lower band edges predominantly controlled by the equivalent mass *M* and the effective elastic coefficient $\bar{K}$, respectively. In the two stable states of the ADM unit, the displacement of the cylinder along the Z axis results in a shift in the center of mass, accompanied by inhomogeneous displacements generated during vibration. These factors lead to distinct band structures in different stable states. Furthermore, it is shown that the Young's modulus *E* and Poisson's ratio ν of the dome structure significantly affect the bandgap characteristics of the ADM unit (see Section S1).

Based on the Brillouin zone of the ADM, Floquet–Bloch conditions were applied to the ADM unit (see Figure 2a(v) and Section S7), the band structures of the peak and valley states were calculated, as shown in Figure 2b(i,ii). The pink and blue shaded regions denote the bandgaps of the peak and valley states, respectively. Within the frequency range of 0–200 Hz, the large‐scale band structure primarily exhibits flat band characteristics, as indicated by the marked points *A*, *B*, *C* and *D*. The corresponding vibration modes are illustrated in Figure 2c. At points *A*, *B*, *C*, and *D*, the vibration amplitude of the substrate is significantly lower than that of the dome shell and resonant cylinders, approaching nearly zero. Taking point *A* as an example, both the valley state and peak state exhibit in‐plane transverse vibration characteristics for the dome and cylinder, while the substrate's vibrational response remains negligible. At the point *D*, the cylinder demonstrates torsional vibration modes in the peak state and transverse vibration modes in the valley state, with similarly insignificant substrate vibration. These observations indicate that the wave energy is mainly localized in the dome and cylinder components, which is characteristic of a typical local‐resonance mode. Collectively, these findings suggest that the natural frequencies across all directions are essentially consistent.

On the non‐flat branch, the dome‐cylinder structure exhibits a distinct longitudinal vibration mode at point *B*. Notably, the flat band behavior undergoes rapid attenuation for wave vectors approaching the Γ. The vibrational mode *B1* in Figure 2c shows that the response is dominated by the longitudinal vibration of the cylinder, while the substrate also exhibits pronounced longitudinal vibrations. This coupling causes efficient elastic wave propagation within this frequency range, contrasting with the localized behavior observed at point *A*. The difference in bandgap characteristics between the valley and peak configurations mainly arises from the distinct influences of the resonators on the structural center of mass within the acoustic dome. This contrast is particularly pronounced in the band corresponding to point *C1*. In the peak state, the strong vibrations are largely confined to the resonators and the dome structure, while energy transfer to the substrate is minimal. In contrast, in the valley state, the mass of the resonators is positioned closer to the substrate along the Z direction, which significantly excites substrate vibrations. Consequently, this branch exhibits a non‐flat dispersion feature. This observation confirms that the emergence of differential bandgaps between the two states is highly dependent on the vertical mass distribution of the resonant elements. The main bandgap of the peak state is 77.4–272.5 Hz. The valley state exhibits the characteristic of dual main bandgaps: 69.7–132.89 Hz and 145.9–273.5 Hz. The suppression of low‐frequency vibrations is a challenging issue in engineering. The characteristics exhibited by the structure in both states demonstrate its superiority in low‐frequency vibration suppression, thereby proving the design's effectiveness in low‐frequency applications.

A finite periodic lattice consisting of 10 × 5 unit cells was constructed for the transmission experiments (see Figure S10a). To minimize the influence of finite boundaries, the base plate was designed with sufficiently large dimensions. As shown in Figure 2d–f, a swept sinusoidal signal $A_{in}\left(f_{e}\right)$ with a frequency range of 10–500 Hz and an amplitude of 3 V was applied to the left end of the specimen using a vibration exciter, providing out‐of‐plane excitation along the Z direction. The excitation signal was generated by a signal generator, amplified by a power amplifier, and then transmitted to the vibration exciter to drive the specimen. Under constant voltage input, the vibration amplitude generated by the exciter exhibits an inverse relationship with the excitation frequency. The output signal $A_{out}\left(f_{e}\right)$ was recorded at the right end of the specimen. The vibration response of the structure was measured using a scanning laser Doppler vibrometer, and the transmission coefficient was calculated according to $T=20\log(A_{out}/A_{in})$. Similarly, in the numerical simulations, harmonic displacement excitation was applied at the input boundary to generate elastic waves in the finite periodic lattice consisting of 10 × 5 unit cells (see Figure 2a(vi)). It should be noted that the present ADM specimen represents a finite lattice, and therefore minor boundary effects may occur near the edges. In the numerical simulations, perfectly matched layers (PMLs) were employed to absorb outgoing waves and suppress boundary reflections.

The experimental result for the peak state is illustrated in Figure 2g(i). The measured attenuation band ranges from 75.04 to 281 Hz, which agrees well with the simulated bandgap in Figure 2f(i). Under identical experimental conditions, the specimen was mechanically programmed into the valley state, and its acoustic response was measured, as shown in Figure 2g(ii). Two attenuation bands were observed experimentally, spanning 49 to 142 Hz and 147 to 244 Hz, respectively, which correspond well to the simulated bandgaps in Figure 2f(ii). The transmission coefficient curves accurately capture both the range and number of bandgaps, and clearly illustrate the differentiated acoustic responses exhibited in the peak and valley states. These results confirm that the experimental setup is capable of reliably characterizing the dynamic response of the ADM and validating the predicted state‐dependent bandgap behavior.

As an illustrative example, under a 145 Hz excitation, the structure exhibits transmission behavior in the valley state, while showing strong attenuation in the peak state. The attenuation difference between the two states exceeds 20 dB. This pronounced state‐dependent transmission contrast demonstrates that elastic wave propagation can be selectively enabled or suppressed through mechanical state switching at frequencies exhibiting acoustic dissimilarity.

Collectively, these results establish the physical basis of MAI‐enabled wave manipulation, in which bistable mechanical reconfiguration governs the acoustic band structure and transmission characteristics of the metamaterial.

### 2.2 Programmable Elastic Waveguiding and Vibration Suppression

Building upon the state‐dependent transmission characteristics established above, elastic wave propagation in the ADM can be spatially controlled by selectively encoding the bistable states of individual units. The reconfiguration of the ADM is enabled by the intrinsic bistability of each dome unit, which allows reversible switching between the peak and valley states under localized mechanical triggering. Accordingly, transitions between different programmed tasks are achieved through selective reprogramming of target units, rather than through spontaneous global recovery. In the present experiments, this process was manually implemented, and the original state was restored through mechanical triggering by applying a reverse stress, as shown in Figure S1b and Movie S3. Each bistable configuration (valley or peak) corresponds to a distinct local transmission or attenuation response, which collectively determines the propagation pathways of elastic waves within the lattice. By selectively switching the states of the dome units, prescribed propagation paths can be directly written into the metamaterial, enabling dynamic reconfiguration of elastic waveguides in real time. This capability allows elastic wave energy to be guided, redirected, or blocked on demand through purely mechanical programming. The overall programming speed is mainly determined by the speed of manual pressing and the number of units to be programmed, although the state transition of an individual bistable unit itself occurs rapidly once the critical snap‐through threshold is reached.

**FIGURE 3:**
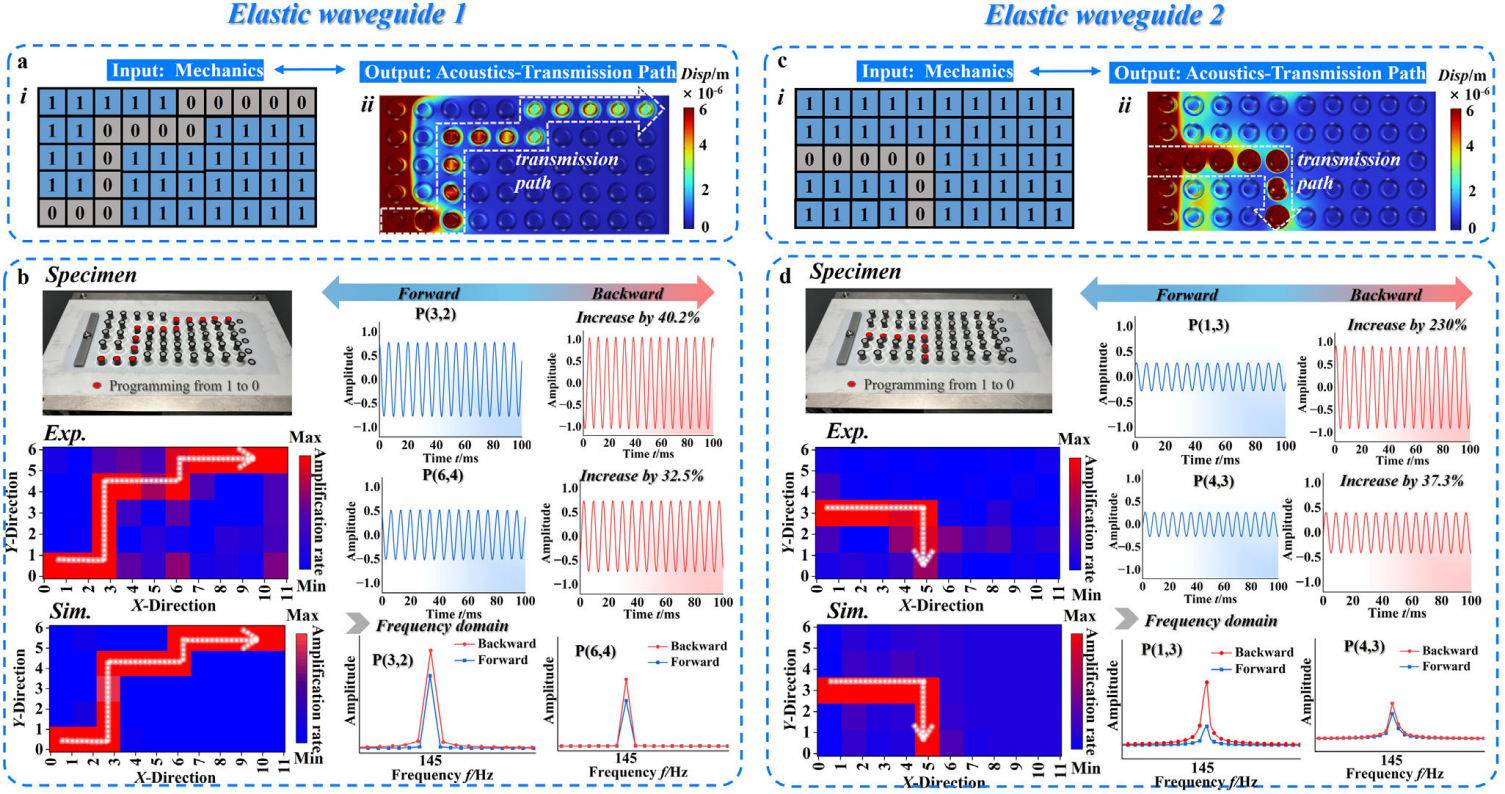
Mechanical programming for elastic waveguidings. (a,c) Finite element simulations illustrating the vibration energy distribution of elastic waves in the programmable ADM. Two distinct waveguiding paths are realized by mechanically presetting the bistable states of individual domes into different binary configurations. (b,d) Experimental demonstrations at 145 Hz, achieved by locally switching selected units from state “1” to “0”. Photographs of the corresponding waveguide specimens are provided, together with representative unit responses highlighting the distinct behaviors before and after programming in both the time and frequency domains.

The programmable acoustic routing was validated through finite‐element simulations and experimental measurements on the ADM composed of 10×5 units (see Sections S5 and S7). The bistable states of individual domes were mechanically preset to form binary maps (Figure 3a(i),c(i)), defining specific propagation routes for elastic waves. When the bistable units along a predefined path were switched from the “1” state to the “0” state through mechanical actuation, the simulated displacement fields showed that 145 Hz elastic waves were strongly confined to the programmed path, while the surrounding “1” state regions suppressed wave transmission because of their stop‐band characteristics (Figure 3a(ii),b(ii)).

Under experimental conditions consistent with the finite element simulations, the high damping of the material and the limited propagation distance of the medium cause the absolute displacement amplitude to primarily reflect the overall attenuation trend with increasing distance from the excitation source. This effect may obscure the programmable wave manipulation behavior of interest. To avoid ambiguity in the interpretation of the results, we introduced a relative vibration response metric to visualize the results of the experiment, which normalizes the measured response with respect to a reference state. For a unit located at position L(x,y), the relative vibration response metric λ is defined as $\lambda \left(x,y\right)=\frac{A_{\text{post}}-A_{\text{pre}}}{A_{\text{pre}}}$, where $A_{\text{pre}}$ and $A_{\text{post}}$ denote the vibration amplitudes before and after programming, respectively. A positive value of λ indicates amplification rate, whereas a negative value indicates attenuation rate. This representation enhances the contrast between different programmed transmission paths. To improve the consistency between experimental results and numerical predictions, the same processing is applied to the simulation results. The results are shown in Figure 3b,d. Consistent with the simulation, the bistable units along a predefined path were switched from the “1” state to the “0” state through localized manual pressing. The experimental results exhibited the same programmable waveguiding behavior at 145 Hz. Elastic waves followed the predesigned routes with significantly enhanced transmission along the paths. This effect originates from the state‐dependent band structure differences introduced by the MAI mechanism. Outside the programmed paths, a small amount of elastic wave scattering and reflection is present, however, material damping—particularly from the silicone dome—leads to rapid attenuation of reflected waves, and, together with normalization relative to a reference state, further reduces their visibility. Therefore, although some reflections exist, they do not dominate the overall response compared to the waves propagating along the programmed paths.

In addition, time‐ and frequency‐domain responses of several units before and after programming are presented, showing a maximum transmission increase of 230% relative to the regions outside the programmed path.

**FIGURE 4:**
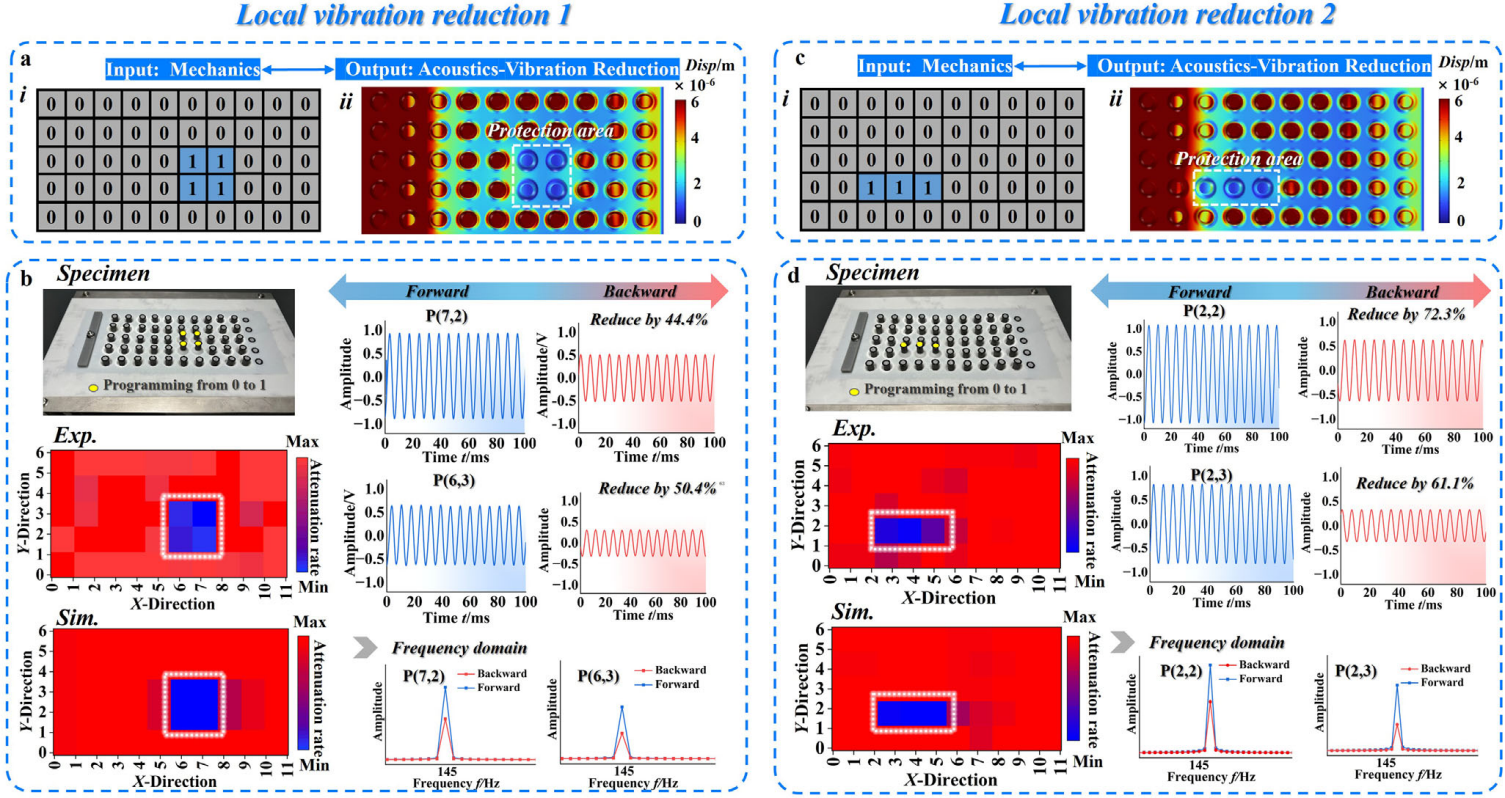
Mechanical programming for local vibration attenuation. (a,c) Finite element simulations showing the spatial distribution of vibration energy, where localized attenuation zones are achieved by mechanically presetting the bistable states of individual domes into designed binary maps. The programmed regions exhibit significantly reduced vibration compared to the surrounding areas. (b,d) Experimental validations at 145 Hz, realized by locally switching selected units from state “0” to “1”. The corresponding specimens are presented, along with representative unit responses demonstrating the pronounced attenuation before and after programming in both the time and frequency domains.

Beyond programmable acoustic routing, the same MAI principle can also be extended to achieve localized vibration suppression. As shown in Figure 4a,c, vibration isolation is realized by configuring selected protective zones in the peak state, while maintaining the surrounding non‐protective units in the valley state to permit wave transmission. Finite‐element simulations indicate that the vibration amplitude inside the protected area decreases to less than 2% of that of the incident wave, confirming the strong shielding capability. When the local mechanical states are programmed in this manner, the ADM forms a barrier‐like region that redirects vibration away from the protected area.

The corresponding experimental results exhibit excellent agreement with the simulations (Figure 4b,d). By programming the local units from “0” to “1”, the maximum transmitted energy is attenuated by 72.3%, demonstrating efficient and reversible vibration isolation at 145 Hz. These results highlight the versatility of the MAI‐based metamaterial, which can not only route acoustic waves along programmable pathways but also selectively suppress local vibrations in a targeted manner.

The discrepancies between the experiments and the finite‐element simulations mainly arise from the difference between the idealized numerical model and the complexity of the actual structure (see Section S9, for detailed analysis).

Overall, these results demonstrate that spatially encoding bistable mechanical states enables programmable control of elastic wave propagation and attenuation within the ADM. By mechanically switching the local configurations of the dome units, the same lattice can be reconfigured on demand to function either as an elastic waveguide or as a localized vibration attenuation structure. Moreover, the corresponding propagation pathways and attenuation regions remain fully reassignable through mechanical state switching, providing a flexible platform for elastic wave manipulation.

In the present experiments, the bistable states were programmed through manual pressing. This approach is consistent with the intended application scope of this work. The state‐switching process could be further extended to externally assisted programming strategies, such as motor‐driven arrays [21], pneumatic‐driven arrays [39], or other programmable mechanical loading systems. Under such extended implementations, the overall programming speed would be mainly determined by the specific actuation mode and system integration strategy. In particular, the adaptive one‐press programming strategy introduced in Section 2.4 provides one possible route toward rapid, repeatable, and large‐area reprogramming, thereby significantly improving programming efficiency and scalability compared with element‐by‐element manual operation.

### 2.3 Programmable Defect States for Energy Harvesting

In addition to programmable waveguiding and vibration attenuation, reconfiguring local bistable states can also induce controllable defect modes in the metamaterial.

In phononic and elastic wave metamaterials, defect states arise when the periodicity of the lattice is intentionally broken, producing localized vibration modes within the bandgap. These modes confine energy around the defect sites, which offer a powerful approach for elastic energy localization [40, 41, 42, 43]. When coupled with piezoelectric transducers, such modes can capture ambient vibrations and convert them into electrical energy, enabling long‐term self‐powered operation of electronic devices. To extend the usable bandwidth of energy localization, various multi‐defect configurations have been explored, such as dual defects [44], line defects [45], and L‐shaped defects [46]. However, conventional defect engineering typically relies on permanent structural modification or external stimuli, which limits reconfigurability.

In contrast, the bistable ADM proposed here provides a programmable route to realize such defect states mechanically. Each bistable unit can act as a “defect generator”, where its state transition modifies the local stiffness and mass distribution, thereby altering the effective band structure. By selectively programming the spatial distribution of these defects, energy localization can be induced in specific regions of the ADM at targeted frequencies. This programmable defect state mechanism establishes a direct physical link between mechanical configuration and energy trapping, offering a new route toward energy management.

**FIGURE 5:**
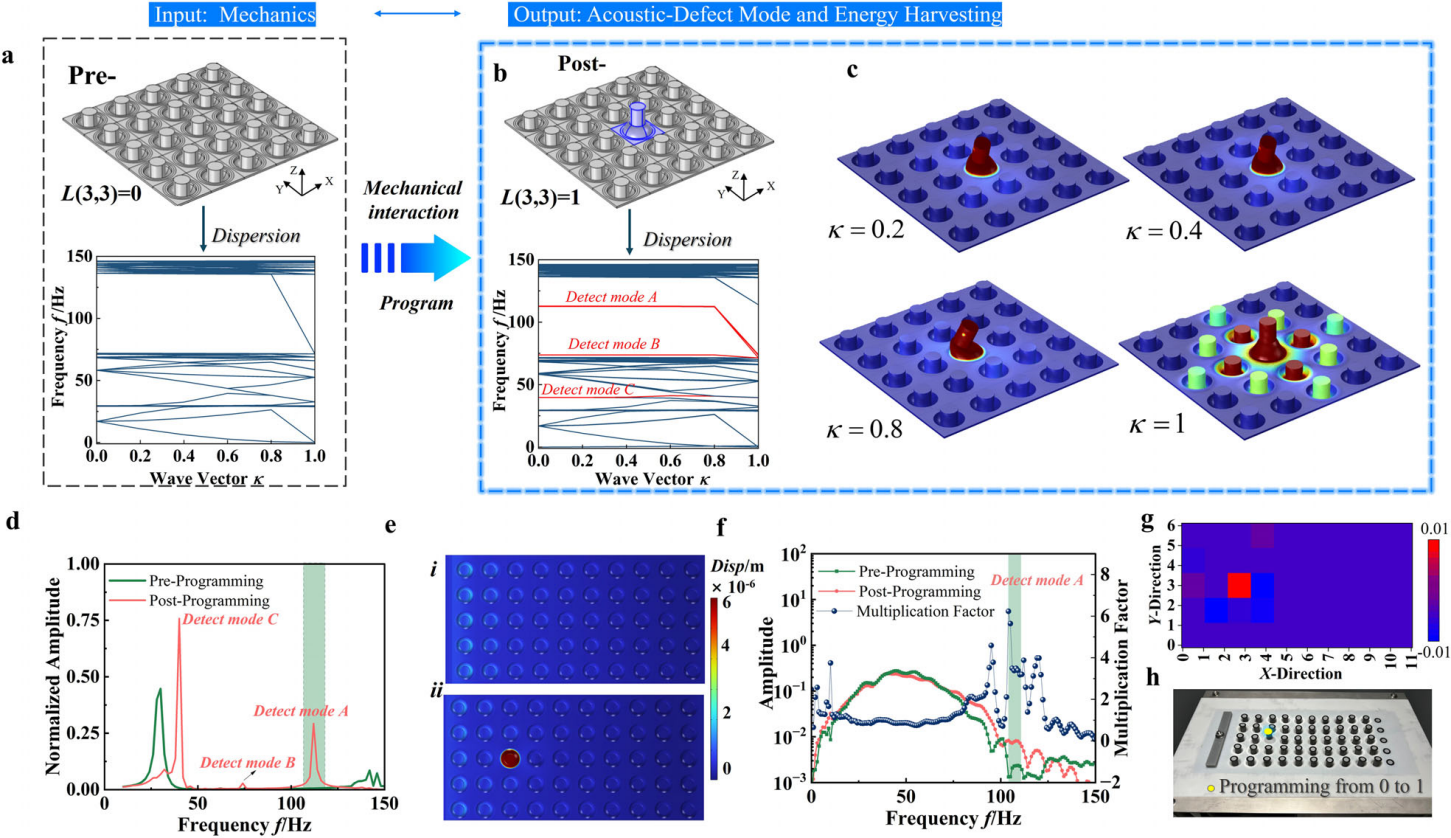
Interaction between mechanical programming and acoustic defect states for energy harvesting. (a) Supercell structure and corresponding band structure before programming (L(3,3) = 0). (b) Supercell structure and band structure after programming (L(3,3) = 1), with red markers indicating the emergence of defect modes. (c) Vibrational modes localized within defect mode A. (d) Amplitude‐frequency response of L(3,3) before and after programming. (e) Measured amplitude at the defect with frequency 112 Hz: (i) before and (ii) after programming. (f) Experimental amplitude‐frequency response demonstrates the emergence of the point‐defect mode. The frequency‐dependent enhancement factor accurately identifies the resonance peak of defect mode A. (g) Experimental measurement of energy localization within (h) the point‐defect specimen.

A perfect periodic structure (L(3,3) = 0) was first constructed, and the band structure of a 5 × 5 supercell was computed via finite element simulations (Figure 5a). The unit L(3,3) was then programmed to “1” to introduce a point defect, and the band structure of the modified supercell was recalculated. Three distinct defect bands emerged at 112.65 Hz (Defect Mode A), 73.73 Hz (Defect Mode B), and 39.77 Hz (Defect Mode C), as shown in Figure 5b. The vibration characteristics of defect mode A are further examined in Figure 5c. At κ=0.2 and κ = 0.4, the defect unit exhibits a torsional mode with energy strongly confined within the dome‐cylinder system, while the surrounding units remain nearly motionless. When κ=1, the energy gradually diffuses outward from the defect center. Although the adjacent valley‐state units (L(3,2), L(2,3), L(3,4), L(4,3)) display slight longitudinal motion, the defect cell consistently maintains the highest energy concentration. These results confirm that, despite the defect band not being perfectly flat, pronounced energy localization is still achieved, validating the effectiveness of the proposed defect mode strategy. To quantitatively assess the energy localization effect, a periodic structure was subjected to 10–300 Hz broadband excitation, and the amplitude frequency responses of L(3,3) before and after programming were compared (Figure 5d). After introducing the defect, pronounced peaks appear at 112, 74, and 40 Hz, corresponding well to the simulated defect bands in Figure 5b, with a deviation below 2.1%. Taking defect mode A as an example, the amplitude at 112 Hz increases by a factor of 27 after programming, demonstrating substantial energy concentration (Figure 5e). The experimental results further validate this behavior (Figure 5f). Slight deviations are observed, mainly due to the non‐uniform spectral amplitude of the broadband excitation. Beyond comparing the amplitude variations pre‐ and post‐programming, we quantified the enhancement factor to unambiguously characterize the defect states. Defect mode A is distinctly resolved with a relative error of less than 3.5%. Because of the finite periodicity of the specimen, the low‐frequency modes (B and C) were therefore excluded from further consideration. Finally, a 112 Hz sinusoidal excitation was applied, and the relative displacement differences among the unit cells before and after programming were used to characterize the localization behavior (Figure 5g,h), demonstrating that the L(3,3) = 1 unit exhibits a distinct energy confinement.

Collectively, these results demonstrate that by actively controlling the state transition of ADM units, defect states can be programmed at arbitrary L(x,y) positions within the same ADM structure, enabling interactive coupling between mechanical actuation and acoustic energy harvesting.

**FIGURE 6:**
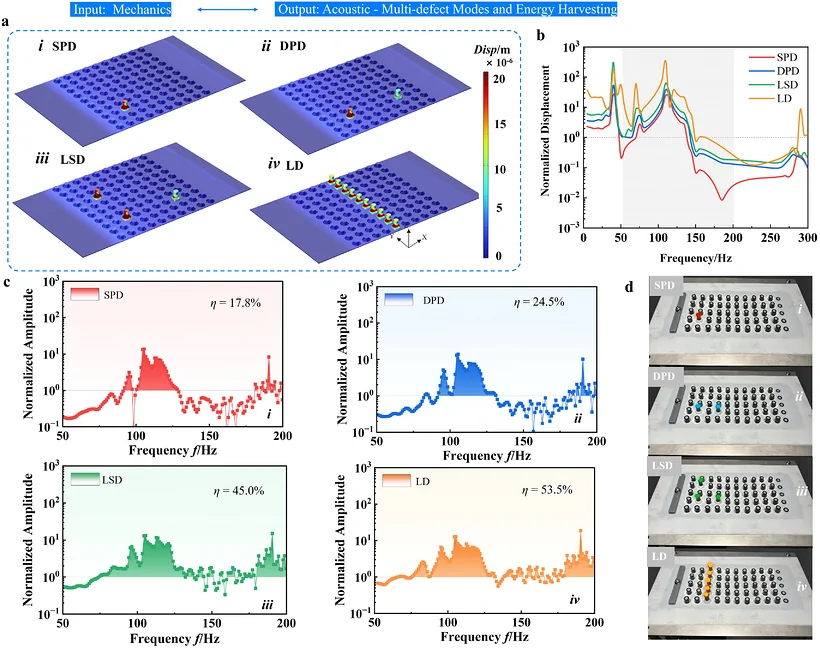
Interaction between mechanical programming and acoustic multi‐defect states for energy harvesting. The investigated configurations include: (i) single point defect (SPD), (ii) double point defect (DPD), (iii) line shaped defect (LSD), and (iv) liner defect (LD). (a) Demonstration of various multi‐defect modes within a single ADM. (b) Frequency‐dependent vibration amplitudes for different multi‐defect modes. (c) Experimentally measured amplitude‐frequency responses. (d) Structural configurations of the multi‐defect specimens.

Extending this concept to multi‐defects, the mechanical programming of their spatial arrangement allows controllable coupling between defects, forming hybridized energy wells with tunable energy localization characteristics. As illustrated in Figure 6a, taking the Single‐Point Defect (SPD) configuration as a baseline, Double‐Point Defect (DPD), L‐Shaped Defect (LSD), and Linear Defect (LD) modes exhibit distinct energy localization patterns. A quantitative comparison of their frequency‐dependent localized amplitudes is presented in Figure 6b, where the responses of multi‐defect modes are represented through amplitude superposition. Numerical simulations confirm that multi‐defect modes can be reconfigured on demand, resulting in significant variations in localization intensity and effective bandwidth. These trends are further corroborated by the experimental observations in Figure 6c.

For the samples exhibiting four programmed multiple‐defect states (Figure 6d), the energy accumulation at the defect units was evaluated using normalized amplitudes. The fraction of the frequency bands η with an amplitude enhancement factor N>1 (see Section S4) relative to the total frequency range is highlighted as the shaded area in Figure 6c. These results demonstrate that programmable multiple‐defect configurations enable controlled energy localization and harvesting within the ADM.

By mechanically encoding bistable states, defect modes and their associated energy localization can be flexibly assigned on demand, highlighting the potential of MAI‐enabled metamaterials for vibration energy harvesting applications.

### 2.4 Shape Adaptive One‐Press Programming Strategy

While the above demonstrations confirm the versatility of MAI‐enabled metamaterials in elastic wave control and energy localization, they primarily rely on sequential, unit‐by‐unit mechanical programming. For practical deployment and large scale reconfiguration, more efficient and user‐friendly programming strategies are highly desirable.

Compared with other types of deformable metamaterials [30, 31, 47, 48], multistable structures are particularly well‐suited for shape adaptation, as they enable reversible transitions between multiple stable configurations without requiring continuous energy input [47, 49]. Leveraging this characteristic, the proposed structure enhances programming efficiency through its shape adaptability and reconfigurability, enabling more versatile and stable morphological transformations.

**FIGURE 7:**
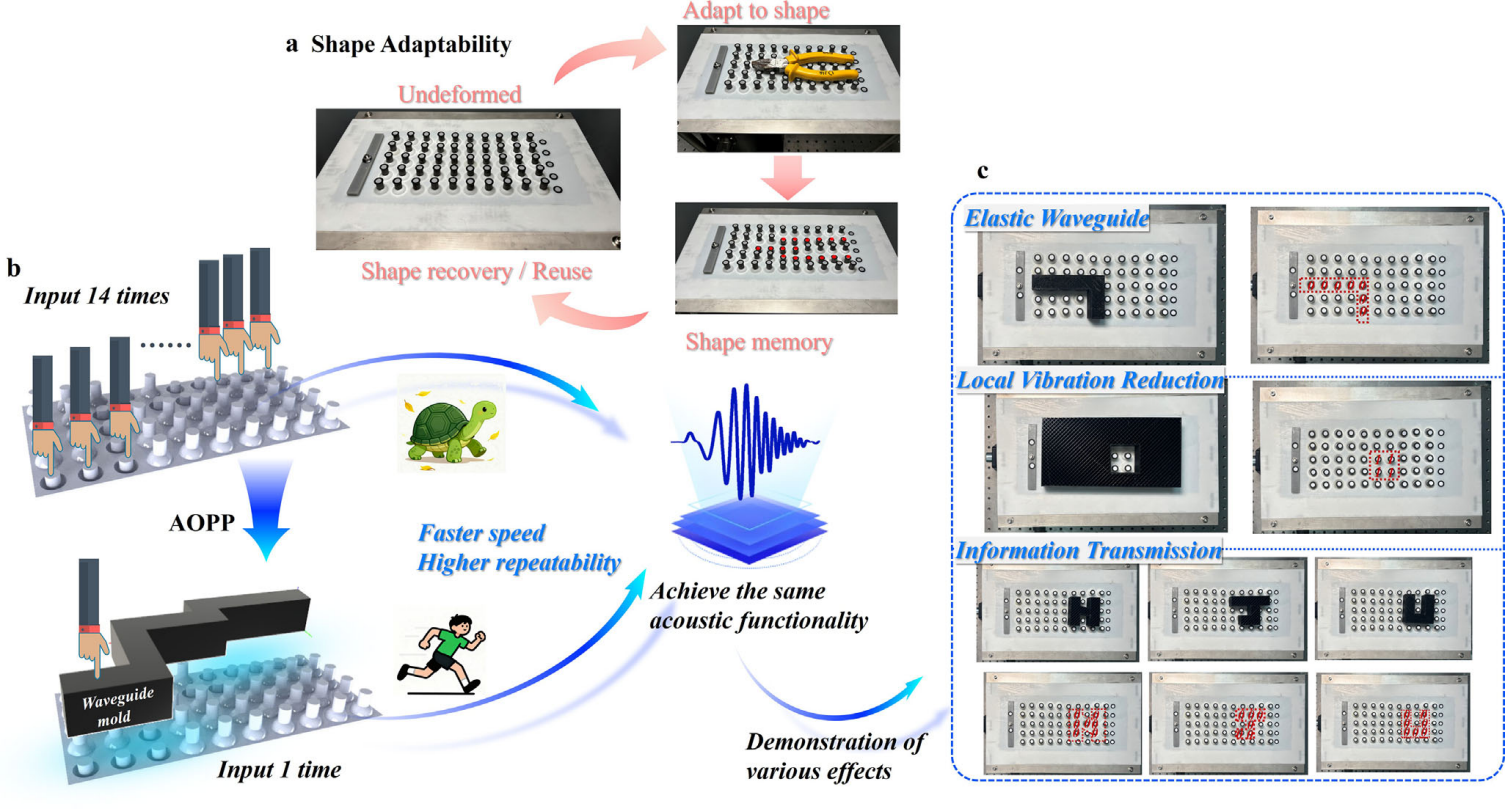
The AOPP strategy for MAI metamaterials. (a)Shape adaptability and reconfigurability of ADM elastic wave metamaterials. When objects of different shapes are placed on the structure, the corresponding units adaptively reprogram from state “1” to “0”, when the object is removed, it exhibits shape memory and repeatability. (b) Compared to conventional MAI programming method, the AOPP strategy demonstrates faster speed and higher repeatability in achieving the same acoustic functionality. (c) Demonstration of the effects achieved through the AOPP.

Figure 7a demonstrates the shape adaptability and reconfigurability of the ADM. When loaded with objects of different geometries, the local units autonomously reprogram their states to conform to the external boundary, while simultaneously exhibiting excellent shape memory and reversible reconfigurability. This inherent morphological adaptability provides a physical basis for more rapid programming control.

Hence, we introduce an adaptive one‐press programming (AOPP) strategy, which enables simultaneous and rapid reconfiguration of multiple bistable units through a single mechanical action. The shape‐adaptive feature discussed here refers to improved programming efficiency enabled by the shape conformability of the bistable architecture under externally applied preset loading patterns, rather than autonomous adaptation to environmental stimuli.

As shown in Figure 7b, models with identical preset acoustic functionalities were fabricated using 3D printing (see Section S8). Taking the elastic waveguide as an example, a single pressing operation can simultaneously trigger large‐scale and coordinated mechanical transformations across the metamaterial array, enabling one‐step programming of its acoustic response, whereas conventional element‐by‐element programming requires 14 individual operations. Likewise, when reconfiguration is required for a subsequent programming task, the structure can be reset to its original state through a single reverse pressing operation by applying the mold from the opposite direction, thereby enabling rapid reprogramming.

Such adaptive morphological coding enables both information encoding and functional reconfiguration within the same physical platform. Figure 7c presents several demonstration results based on the AOPP mechanism, such as elastic waveguide (see Movie S3) and local vibration reduction. Beyond acoustic function, as a proof of concept, 3D printed models with the letters “N” “J” “U” were employed to demonstrate information transmission: by applying the AOPP process, the structural geometry itself serves as a direct carrier of encoded information. The AOPP eliminates the need for element‐by‐element tuning. Through a single press, the entire metamaterial can be globally reprogrammed, achieving rapid, scalable, and reversible functional modulation.

## 3 Conclusion

In summary, this work demonstrates a mechanically programmable metamaterial that leverages mechanical–acoustic interactions to achieve reversible, on‐demand modulation of elastic waves via bistable state switching.

Without relying on electrical, magnetic, or thermal stimuli, the proposed acoustic dome metamaterial provides a physically intuitive and energy‐efficient route toward programmable elastic wave control. A key contribution of this study lies in leveraging bistable mechanical states as reconfigurable functional units, through which elastic wave propagation, attenuation, and defect‐state formation can be selectively programmed at the unit‐cell level. The consistency between numerical simulations and experimental results confirms the effectiveness of mechanical–acoustic interaction as a mechanism for active elastic wave manipulation. Beyond demonstrating multiple programmable functionalities, an adaptive one‐press programming strategy is further introduced to significantly improve programming efficiency and scalability, enabling rapid reconfiguration of complex mechanical state patterns through a single mechanical action.

At the current stage, the proposed framework is mainly suited to scenarios in which the target wave functionality is predefined or only infrequently updated, while its programming speed and real‐time adaptability remain limited. Owing to the strong structural adaptability of the proposed platform, future work may further integrate external actuation or sensing strategies to improve programming speed and functional intelligence.

Overall, this work establishes mechanical–acoustic interaction as a robust, physically grounded framework for elastic wave programming, offering a versatile and scalable platform for the next generation of reconfigurable acoustic systems.

## 4 Experimental Section

### 4.1 Sample Fabrication and Design

The specimen matrix material is polylactic acid, fabricated using light‐curing 3D printing ($\rho =1200\;\mathrm{kg}/m^{3}$, E=2681MPa). The shell structure is made of soft rubber material (DPI 8400) produced through a mold replication process ($\rho =1140\;\mathrm{kg}/m^{3}$, E=0.66MPa, ν=0.4). The resonant cylinder is made of lead, manufactured by lathe machining ($\rho =11343\;\mathrm{kg}/m^{3}$, E=17000MPa). These three components are bonded using a specialized 3D printing adhesive. (see Section S2).

### 4.2 Experimental Setup and Measurement

An integrated experimental setup was established, consisting of a signal generator (TEKTRONIX, AFG 31000), a power amplifier (WXSA, SA‐PA020), and a vibration generator (WXSA, SA‐JZ010) as the excitation source, while the vibration response was measured using a scanning laser Doppler vibrometer (HUAQIN OPTACOUS, PLV‐633) and a data collection card(NI, NI‐9234) with high spatial resolution. Stepping movement platforms (FUYU, FLK40), driven by a displacement platform controller (Zolix, ZC300), were employed to adjust the position of the laser vibrometer, enabling vibration signals to be collected at different locations on the fixed specimens.(see Section S5).

### 4.3 Numerical Simulations

The mechanical compression response of the structure was characterized using the commercial finite element package ABAQUS. Simulations were conducted using a static analysis with C3D10 elements. In the compression model, the sides of the acoustic dome shell were constrained, and a vertical displacement was applied to the top surface.(see Section S6)

The acoustic properties, including the dispersion relations and transmission coefficients, were investigated using COMSOL Multiphysics. For the dispersion analysis, a unit cell was modeled with 3D elements and Bloch periodic boundary conditions within the Solid Mechanics interface. The eigenfrequencies and corresponding mode shapes were obtained via a parametric sweep. To evaluate the transmission characteristics, a finite supercell consisting of a 10×5 array of acoustic domes was constructed. Periodic boundary conditions were applied laterally, and a frequency domain study was performed. The transmission coefficient curve and energy distribution were derived by computing the displacement fields at the input and output boundaries using boundary probes. (see Section S7)

## Conflicts of Interest

The authors declare no conflicts of interest.

## Supporting information

Supporting file

Supplemental Movie 1

Supplemental Movie 2

Supplemental Movie 3

## Data Availability

The data that support the findings of this study are available from the corresponding author upon reasonable request.
