# Supplementary material for: Programmable Elastic Wave Control Via Mechanical‐Acoustic Interaction in Bistable Metamaterials
Source: Adv Sci (Weinh). 2026 Jun 12;13(49):e76005. doi: 10.1002/advs.76005 (PMC13336889; doi:10.1002/advs.76005)
Supplement: Supplementary file 1 [file ADVS-13-e76005-s004.pdf]

Supporting Information

Programmable Elastic Wave Control via Mechanical-Acoustic Interaction in Bistable Metamaterials

Yuanyuan Li Yonghua Yu Chuanqing Chen Rui Xu Yulong He Xin Li Ming-Hui Lu*, Yan-Feng Chen*

Y. Li, Y. Yu, C. Chen, R. Xu, Y. He, M.-H. Lu, Y.-F. Chen
National Laboratory of Solid State Microstructures, Nanjing University, Nanjing 210093, China
College of Engineering and Applied Sciences, Nanjing University, Nanjing 210093, China
Email Address: luminghui@nju.edu.cn
X. Li
School of Mechanical Engineering, Nanjing University of Science and Technology, Nanjing 210094, China
Email Address: xinli@njust.edu.cn
M.-H. Lu
Jiangsu Physical Science Research Center, Nanjing 210093, China

Contents

S1 Theoretical Analysis **2**

S2 ADM Design **6**

S3 Mechanical Performance Testing **9**

S4 Effective Bandwidth for Energy Harvesting in Multiple-Defect States **12**

S5 Experimental System **12**

S6 Mechanic Simulation Parameter Settings **13**

S7 Acoustic Simulation Parameter Settings **13**

S8 AOPP Models **16**

S9 Error Analysis between Simulation and Experiment **17**

This Supporting Information provides additional theoretical analyses, experimental details, simulation parameters, and extended discussions.
A conceptual analogy between human–computer interaction (HCI) and mechanical–acoustic interaction (MAI) is first provided to aid intuitive understanding of the MAI-based programming paradigm. In HCI, discrete human inputs are translated into functional outputs through electronic tools. Analogously, in the proposed MAI framework, mechanically encoded bistable states serve as physical inputs that directly reconfigure elastic wave responses, without relying on electronic or digital control systems.

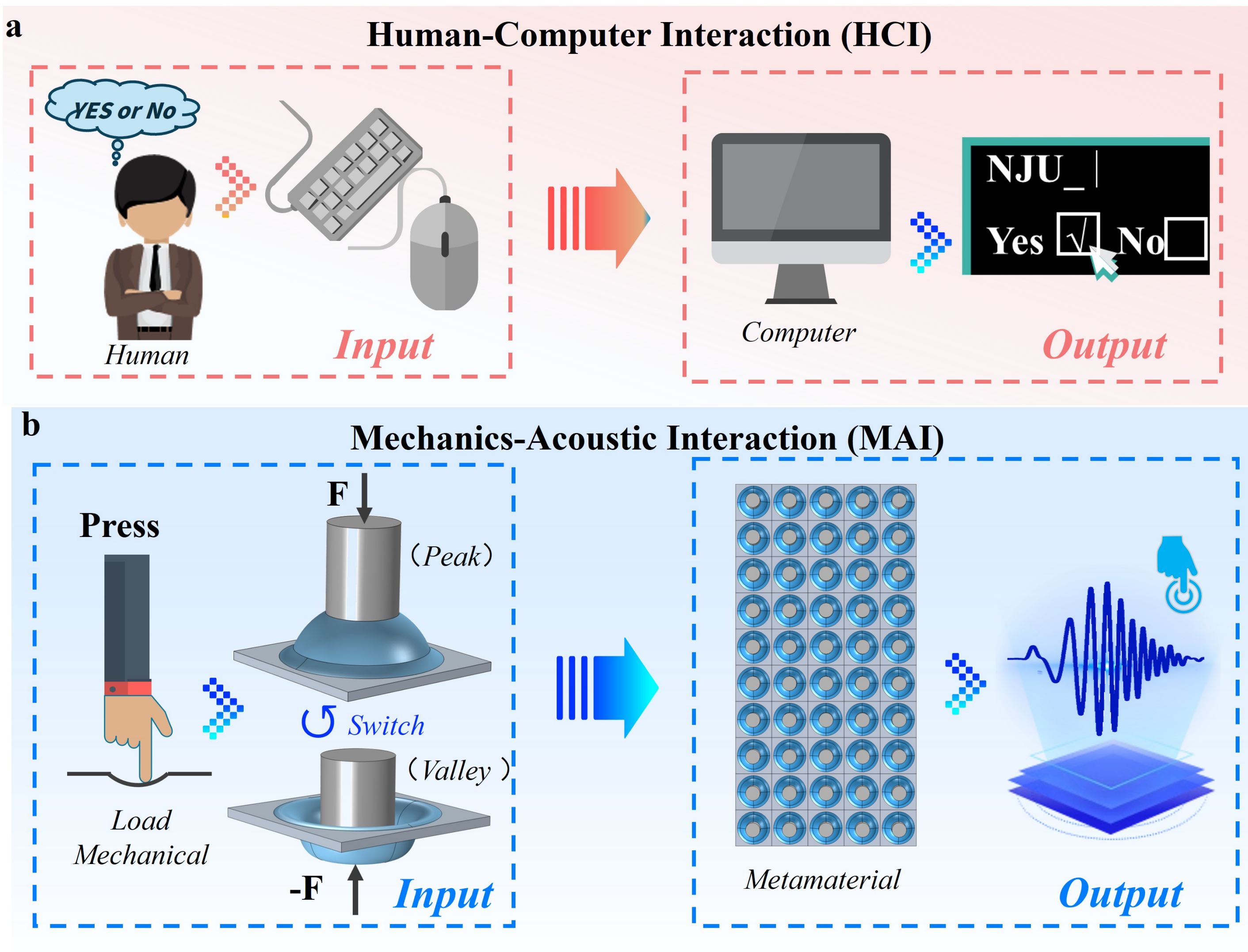

Figure S1: Conceptual analogy between a) human–computer interaction (HCI) and b) mechanical–acoustic interaction (MAI). (This analogy is intended solely to provide intuitive insight into the programming paradigm enabled by MAI and does not imply any direct correspondence between electronic computation and mechanical wave manipulation.)

S1 Theoretical Analysis

In acoustic metamaterials, the mass-in-mass model is a classic local resonance structure. Many researchers have extended it to a two-dimensional mass-in-mass configuration[1, 2, 3, 4, 5], as shown in **Figure S2a**. This configuration serves as a theoretical model for 2D continuum locally resonant metamaterials, as illustrated in **Figure S2b**. In this model, the core is equivalent to the resonant mass m_2, the coating acts as the spring k connecting the m_1 and the m_2, and the hosting matrix is equivalent to the base mass m_1. However, for a 2D continuum unit, the mass of the uniform coating cannot be neglected. Its mass must

be partitioned into two parts, which are respectively assigned to the resonant mass and the host matrix [6, 7]. Let m_{core}, m_{coating}, and m_{matrix} denote the masses of the core, coating, and matrix, respectively. Consequently, the effective masses can be expressed as:

$$m_1 = m_{\text{core}} + \frac{\alpha m_{\text{coating}}}{1+\alpha} \tag{1}$$

$$m_2 = m_{\text{matrix}} + \frac{m_{\text{coating}}}{1+\alpha} \tag{2}$$

where $\alpha = \frac{m_2}{m_1} = \frac{m_{\text{matrix}}+m_{\text{coating}}}{m_{\text{core}}+m_{\text{coating}}}$.

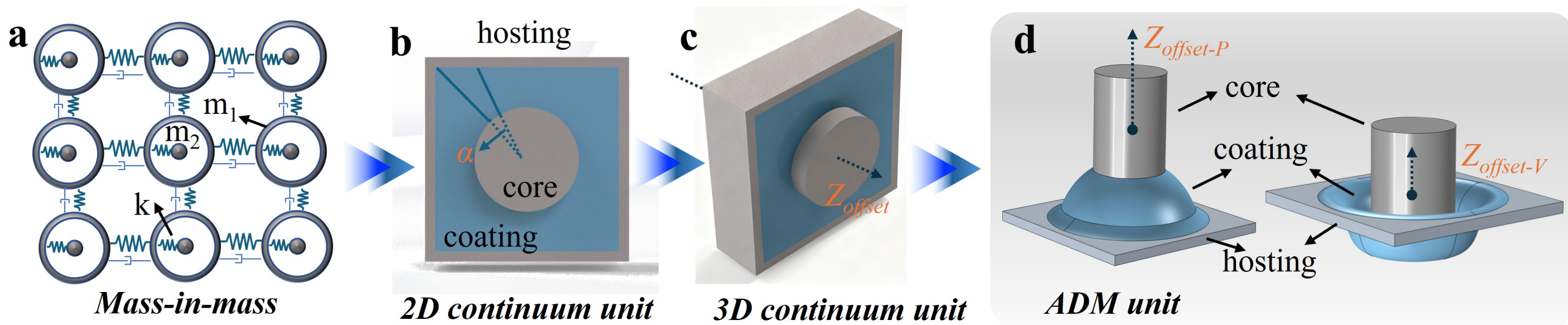

Figure S2: (a) A classical local resonance acoustic metamaterial the mass-in-mass structure. (b) 2D continuous local resonance acoustic metamaterial. (c) 3D continuous local resonance acoustic metamaterial. (d) The Acoustic dome matemeterial (ADM).

The coating is divided into an infinite number of slender units along the wave propagation direction. The central angle of each unit is denoted by θ, and each unit can be regarded as a spring connecting m_1 and m_2. The equivalent stiffness $\overline{K}$ can then be calculated by considering these springs in parallel. The total effective stiffness of the uniform coating is obtained by integrating all tensile and shear stiffness elements over the entire circumference.

$$\overline{K} = \int_0^{2\pi} (dK_t + dK_s) \tag{3}$$

where dK_t, dK_s respectively represent tensile stiffness and shear stiffness.

$$dK_t = C_{11}\frac{r_1 d\theta}{r_2 - r_1}\cos^2\theta \tag{4}$$

$$dK_s = C_{44}\frac{r_1 d\theta}{r_2 - r_1}\sin^2\theta \tag{5}$$

They represent the tensile stiffness and shear stiffness, respectively. r_1 and r_2 denote the inner and outer radii of the coating, while C_{11} and C_{44} are the elastic constants of the coating. For an isotropic elastic coating, the elastic constants C_{11} and C_{44} can be derived from Young's modulus E and Poisson's ratio ν as: $C_{11} = E(1-\nu)/(1+\nu)(1-2\nu)$, $C_{44} = E/2(1+\nu)$. Based on the above derivation, the eigenfrequency formulas for the lower and upper edges of the band gap in 2D continuous local resonant acoustic metamaterials are given by:

$$\omega_1 = \sqrt{\frac{\overline{K}}{m_1}} \tag{6}$$

$$\omega_2 = \sqrt{\frac{\overline{K}}{m_1} + \frac{\overline{K}}{m_2}} \tag{7}$$

According to the aforementioned formulation[6, 7], the bandgap in continuum locally resonant metamaterials is primarily governed by the effective elastic modulus and the effective mass. The effective elastic

modulus is mainly determined by E and ν of the material, while the effective mass is not only influenced by the self-mass of the matrix and the core but also significantly affected by the mass distribution of the coating. It should be noted that the lower edge of the bandgap is dominated by the vibration of the core, whereas the upper edge is governed by the out-of-phase vibration between the core and the matrix.
The 2D continuum structure is extended to 3D, as shown in **Figure S2c**. When the height of the core exceeds that of the matrix/coating, the core position Z_{offset} significantly influences the overall center of mass of the unit cell. This positional variation further markedly affects the core vibration mode, the out-of-phase vibration mode between the core and the matrix, the mass distribution of the coating, and the boundary conditions. To validate this hypothesis, the influence of Z_{offset} on the band structure was investigated using COMSOL finite element simulations, with the results presented in the **Figure S4**.

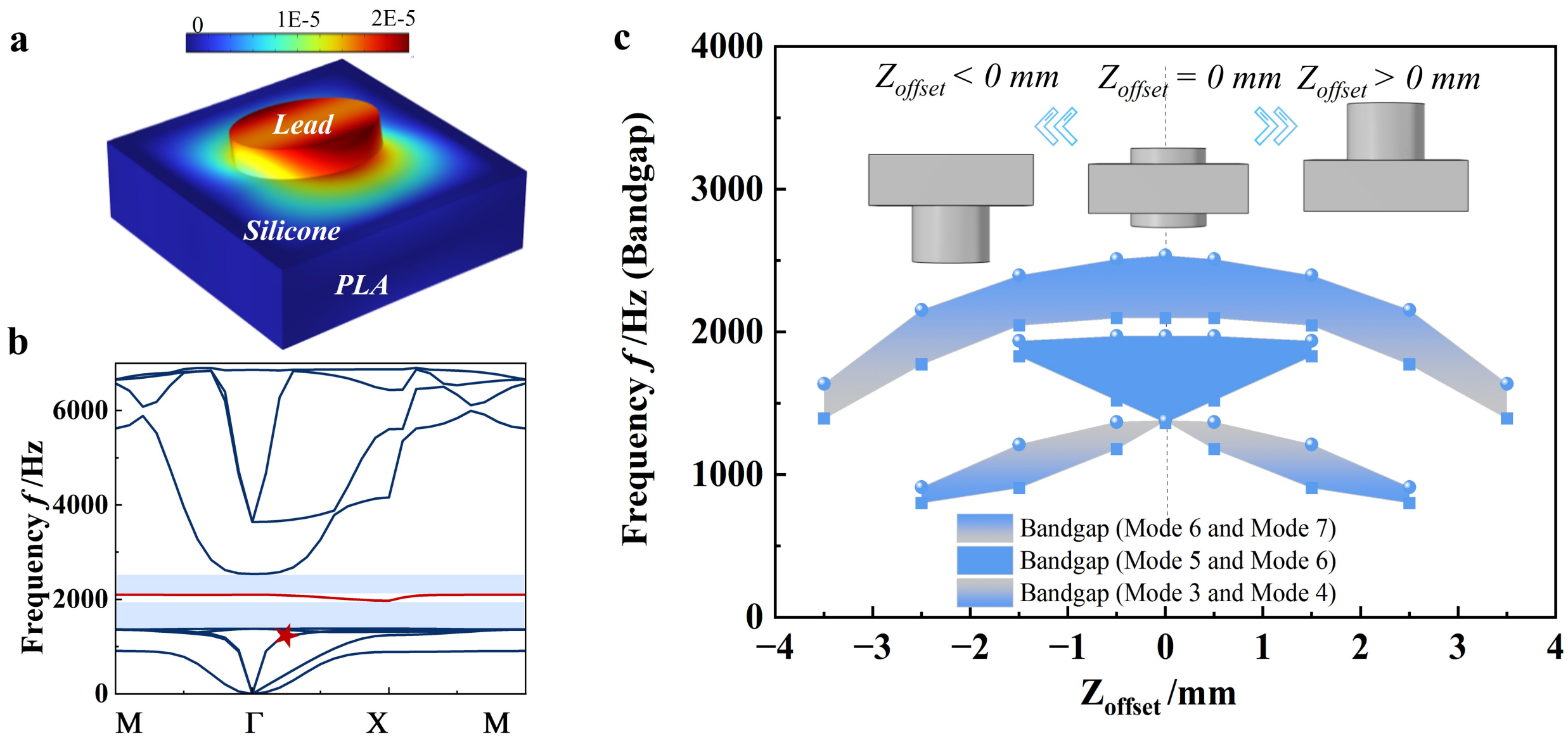

Figure S3: Demonstration of the influence of structural centroid offset on bandgap shift using a typical 3D continuous model. When $(Z_{\text{offset}}) = 0\,\text{mm}$, a) the dispersion curve and b) the vibration mode on κ=1.2, 4th dispersion mode; c) The variation of bandgap with centroid offset Z_{offset}.

The figure verifies the influence of Z_{offset} on the acoustic properties of the three-dimensional continuum locally resonant unit, including vibration modes, band structure, and bandgaps. Based on this, a bistable ADM unit with shell structure was constructed, in which the Z_{offset} of the resonant pillar shows significant differences between the two stable states. According to the aforementioned research, switching between the bistable states enables the acquisition of two acoustic metamaterial units with distinct acoustic characteristics.

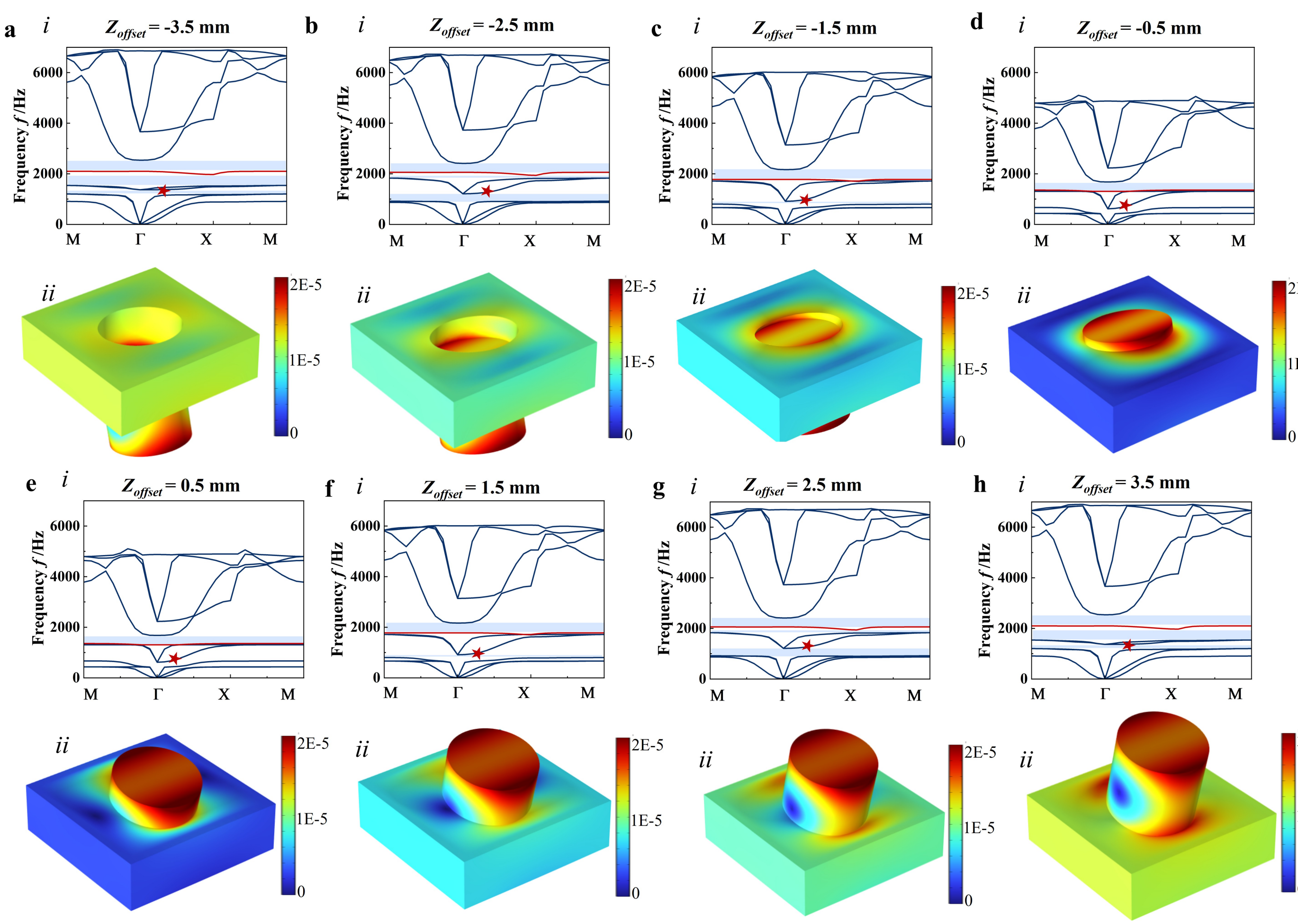

Figure S4: Dispersion curves and vibration modes of 3D continuous local resonance metamaterials with varying centroid offsets. d)–g) When (Z_{offset}) $< 0\,$mm, i)the dispersion curves and ii) the vibration modes on κ=1.2, 4th dispersion mode; h)–k) When (Z_{offset}) $> 0\,$mm, i) the dispersion curves and ii) the vibration modes on κ=1.2, 4th dispersion mode.

S2 ADM Design

Bistable and multistable structures have attracted considerable attention in mechanical metamaterials because they can switch among distinct stable configurations under external stimuli while maintaining the deformed state without continuous energy input[8, 9, 10]. Owing to this unique combination of reconfigurability and state-locking capability, such architectures provide an effective structural basis for programmable mechanical and wave functionalities. In the field of mechanical metamaterials, spherical dome-shaped shells constitute a prototypical three-dimensional bistable structure[11, 12]. Such shells exhibit two mechanically stable equilibria, enabling reversible state switching upon mechanical stimulus.

Figure S5a illustrates the geometric parameters of the shell structure. The Poisson's ratio of the material is ν, When the angle θ is a constant, the threshold parameter $\lambda_d^T(\theta)$ is defined.

$$\lambda_d^T = 1.44\nu + 5.06 \tag{8}$$

The shell's dimensionless parameter λ_d can be expressed as

$$\lambda_d = \left[2(1-\nu^2)\right]^{1/4} \theta \sqrt{\frac{R}{d}}. \tag{9}$$

Theoretical analysis reveals that the structure manifests bistability when its dimensionless geometric parameter falls below a critical threshold:

$$\begin{cases} \lambda_d \leq \lambda_d^T & \text{Monostable} \\ \lambda_d > \lambda_d^T & \text{Bistable} \end{cases} \tag{10}$$

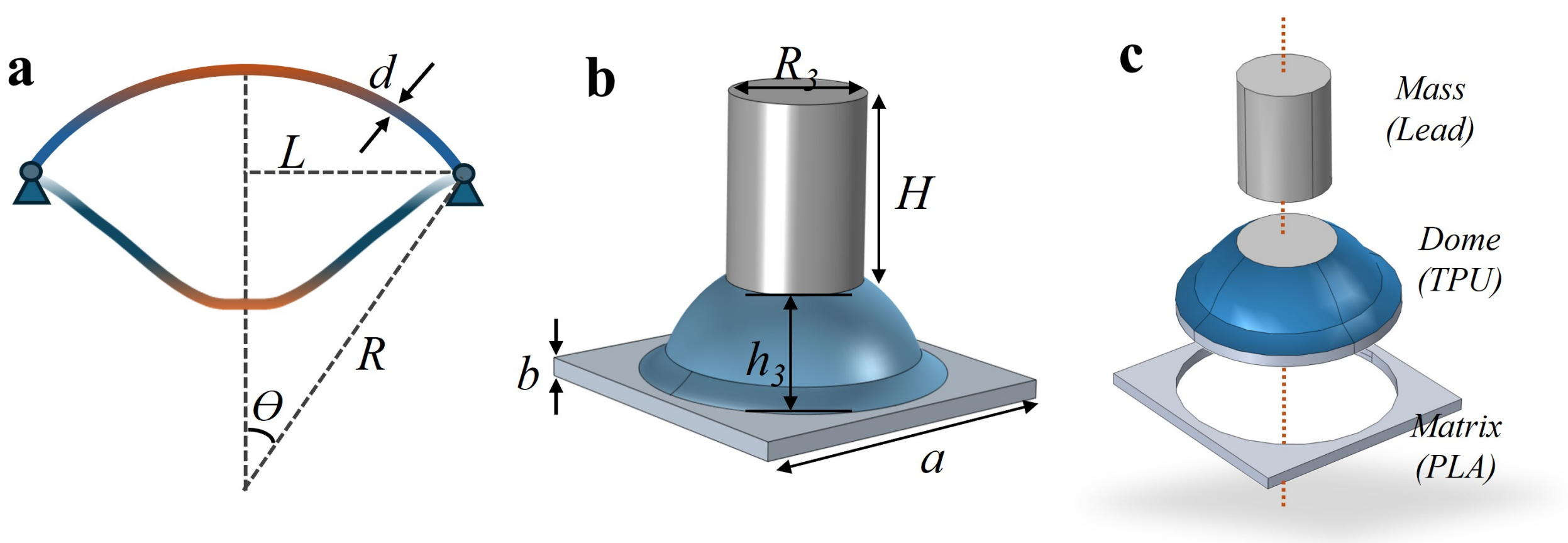

Figure S5: a) Schematic diagram of the bistable structural parameters. b) Schematic diagram of the ADM unit parameters. c) Structural and material composition of the ADM unit.

The ADM unit cell considered in this study possesses the following geometric parameters: The lattice constant is set as a, with the substrate height b, dome shell height h_3, mass column height H and mass column radius R_3. Both the dome shell edge and substrate edge have a width of b, while the dome shell thickness t. The above-mentioned geometric parameter values are shown in the **Table 2** below.

Through calculation, although the shell structure features a flat-cut top, it is hypothesized that the structure would satisfy the bistability condition if it were without this flat-cut top, as illustrated in **Figure S5c**. This condition is given by $\lambda_d > \lambda_d^T$. This hypothesis will be verified via finite element simulation and experiment in next sections.

Furthermore, based on the local resonance theoretical analysis presented in Section S1, the effects of the substrate material and the resonator height on the bandgap characteristics were investigated, as shown in **Figure S6**. It can be observed that as the height H increases, the upper boundary of the bandgap remains

Table 1: Geometry parameters of the ADM unit

Size parameters	Value (mm)
a	20
b	1
h_3	7
H	10
R_3	8
b	1
t	0.7

nearly constant for both the valley and peak states, whereas the lower boundary gradually decreases with increasing H. This trend is consistent with the fundamental relationship between geometric parameters and bandgap formation predicted by the mass-in-mass local resonance model. In addition, the bandgap difference between the two bistable states progressively diminishes.

In addition, the influence of the substrate material on the bandgap characteristics was investigated by comparing two representative cases , as shown in **Figure S7**: PLA and aluminum (Al) substrates. The calculated dispersion relations reveal that the lower boundary of the bandgap remains almost unchanged regardless of the substrate material, indicating that it is primarily governed by the local resonant properties of the pillar–dome assembly rather than the substrate stiffness. However, the upper boundary of the bandgap shifts to a higher frequency when the substrate is made of PLA compared to Al. This observation aligns well with the mass-in-mass local resonance model. Furthermore, the bandgap difference between the two bistable states is found to be only weakly affected by the substrate material.

These parametric studies reveal how the geometric and material configurations govern the formation and tunability of the bandgap, providing essential guidance for designing programmable metamaterials with targeted frequency ranges and adaptive acoustic functionalities.

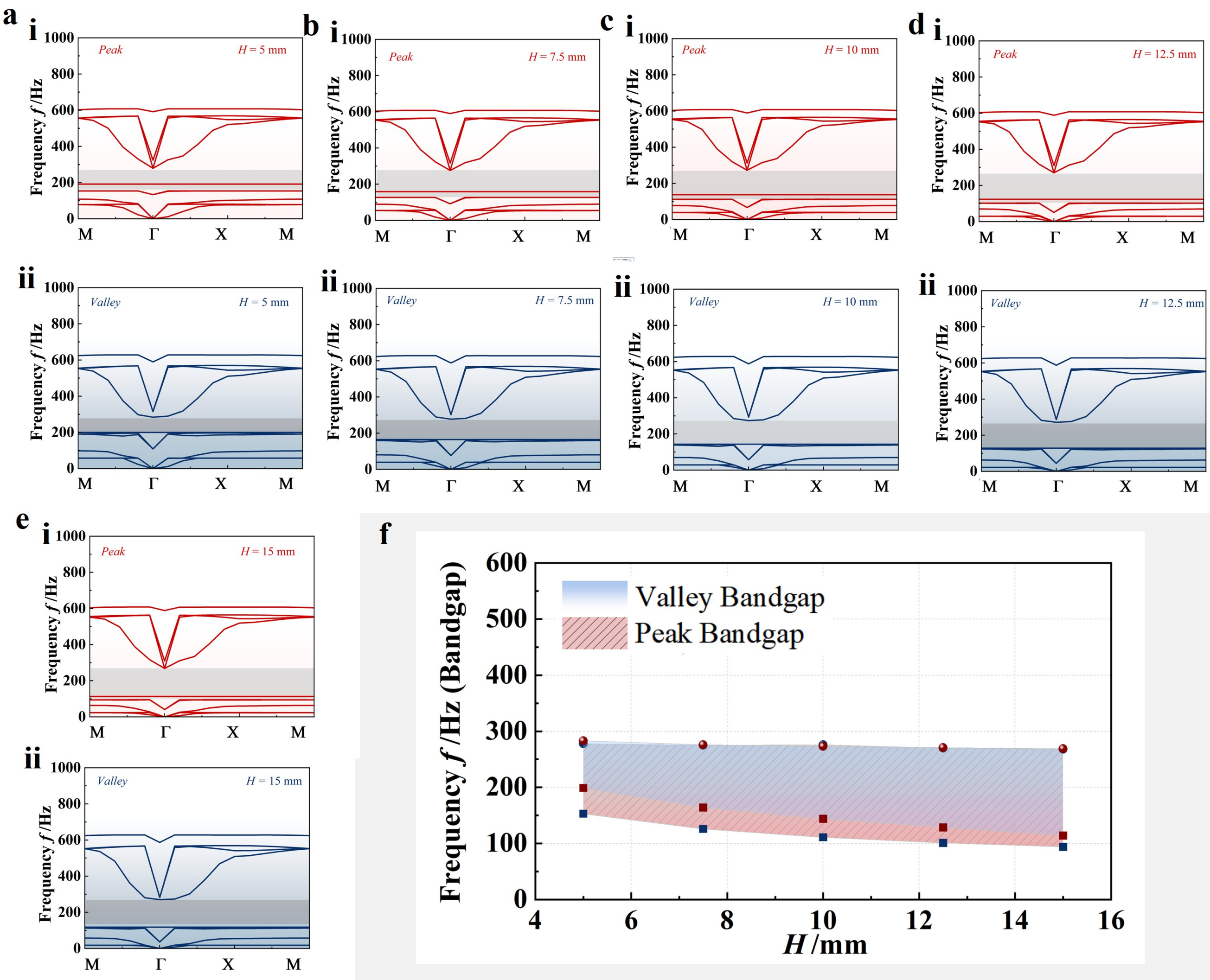

Figure S6: Influence of resonator height on the dispersion characteristics of the programmable metamaterial. a–e) Calculated dispersion relations of the programmable metamaterial at different resonator heights. Panels i) and ii) correspond to the peak and valley states, respectively. The gray-shaded regions indicate the complete bandgaps. f) Dependence of the bandgap frequency range on the resonator height H.

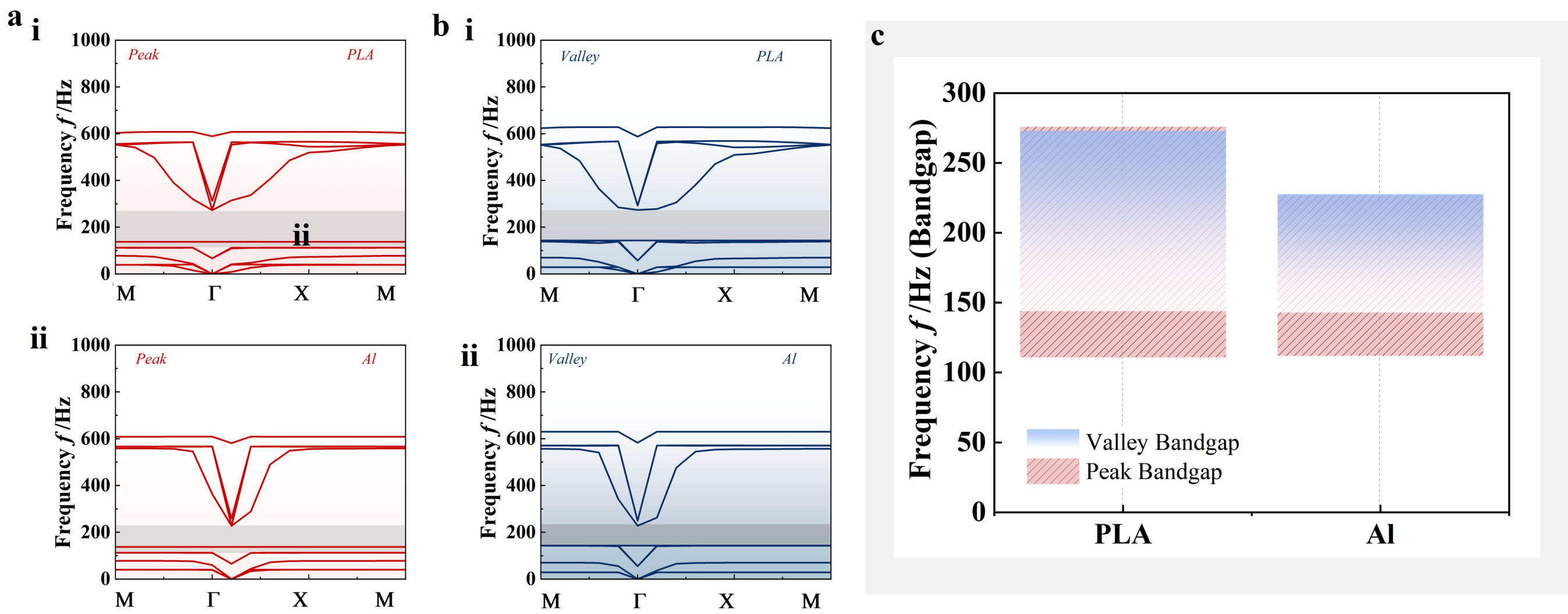

Figure S7: Effect of substrate material on the band structure of the programmable metamaterial. a) b) Simulated dispersion relations of the metamaterial with PLA and Al substrates, respectively. The gray-shaded regions denote the complete bandgaps obtained under both the peak and valley configurations. c) Comparison of the bandgap frequency ranges for the two bistable states under different substrate materials.

S3 Mechanical Performance Testing

Finite element simulations were performed using Abaqus to investigate the mechanical properties of the ADM unit cell. **Figure S8a** shows the displacement-reaction force curve and internal stress distribution. The mechanical behavior of the proposed structures exhibit pronounced negative stiffness and bistable characteristics. Parametric analysis indicated that variations in elastic modulus E and Poisson's ratio ν of the constituent materials significantly influenced the jump threshold of these curves.

The specimen matrix material is PLA, fabricated using light-curing 3D printing ($\rho = 1200\,\text{kg/m}^3$, $E = 2681\,\text{MPa}$). The shell structure is made of soft rubber material (DPI 8400) produced through a mold replication process ($\rho = 1140\,\text{kg/m}^3$, $E = 0.66\,\text{MPa}$, $\nu = 0.4$). The resonant cylinder is made of lead, manufactured by lathe machining ($\rho = 11\,343\,\text{kg/m}^3$, $E = 17\,000\,\text{MPa}$). These three components are bonded using a specialized 3D printing adhesive. The physical specimen was fabricated , exhibiting two stable states(peak and valley), as shown in **Figure S8b**.

Mechanical tests were conducted using the custom tensile fixtures on an universal testing machine (Shimadzu, **Figures S8c,d**), and the experimental results were in good agreement with numerical prediction, the regions I_p and III_p exhibit positive stiffness characteristics, while region II_p demonstrates negative stiffness behavior. When the reaction force $F_r = 0\,\text{N}$, three displacement solutions exist (A, D, E), where A ($x = 0\,\text{mm}$), E ($x = 8.75\,\text{mm}$)correspond to the two stable states of the structure. These mechanical characteristics are critical for the programmability and reconfigurability of the metamaterial: the bistable states serve as switchable configurations, while the negative stiffness enhances the sensitivity of local state transitions, enabling dynamic control of elastic wave propagation at the unit-cell level.

Among the mechanical parameters of shell structures, E and ν not only influence the formation of bistable states and the mechanical response, but also affect the acoustic properties. This relationship is correspondingly explained in Equations (3) to (7). Furthermore, the effect of the elastic modulus E of the constituent material on the bandgap characteristics was systematically investigated to elucidate the stiffness-dependent tuning mechanism of the metamaterial. As shown in **Figure S9**, an increase in E results in a simultaneous upward shift of both the upper and lower band edges, leading to an overall increase in the bandgap frequency range. The relationship between the bandgap boundaries and elastic modulus follows the typical scaling law of the mass–in-mass model. This behavior originates from the enhanced effective stiffness of the local resonant system, which raises the intrinsic vibration frequency of the resonator.

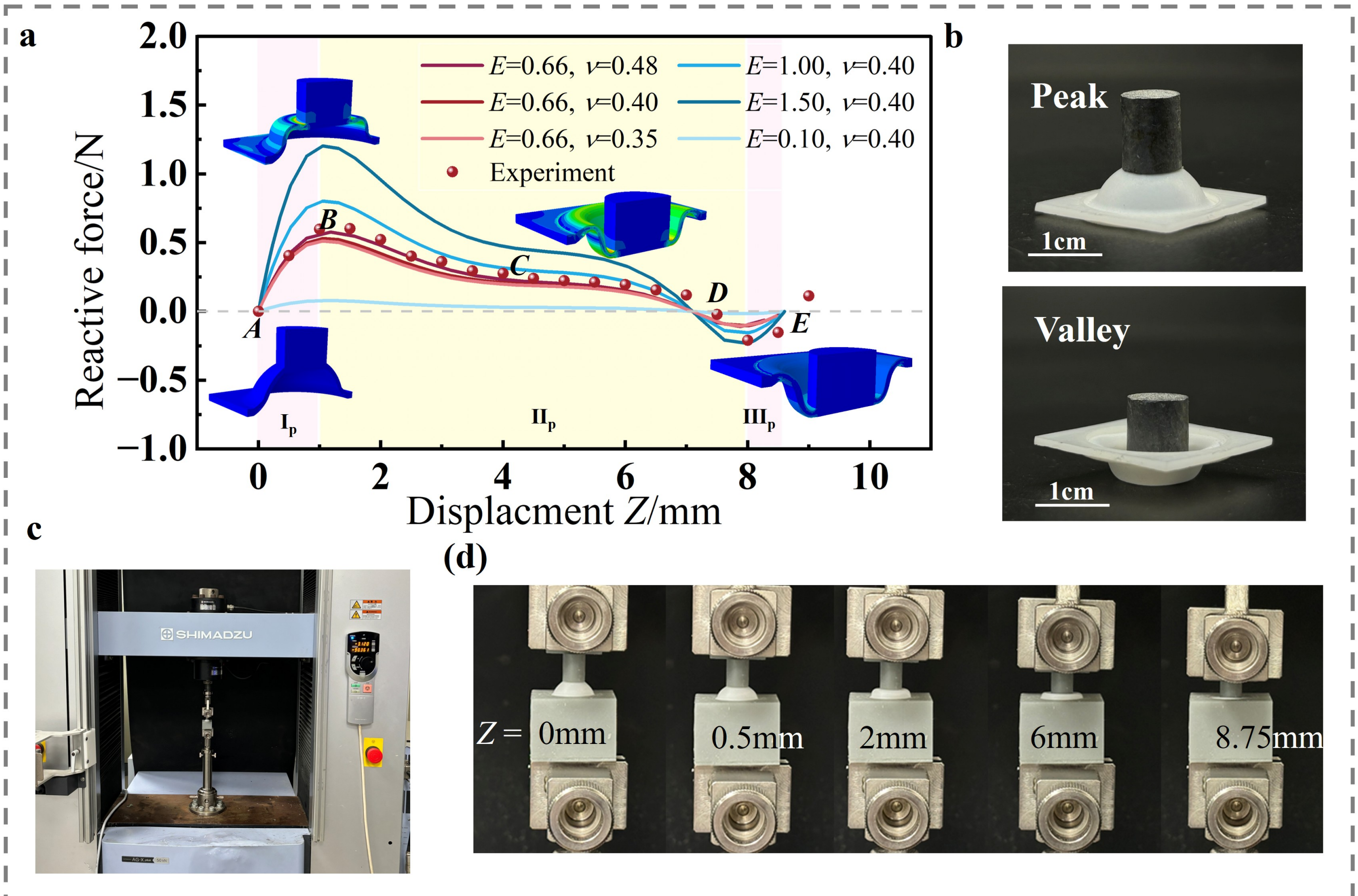

Figure S8: a) The mechanical properties of the ADM unit: the relationship between displacement Z and the reactive force F_r, as well as the corresponding distribution of structural strain energy. b) The ADM unit specimen. c) The universal testing machine (Shimadzu). d) The deformation transition of the specimen from the peak configuration to the valley configuration.(see MOV **S2**, Supporting Information)

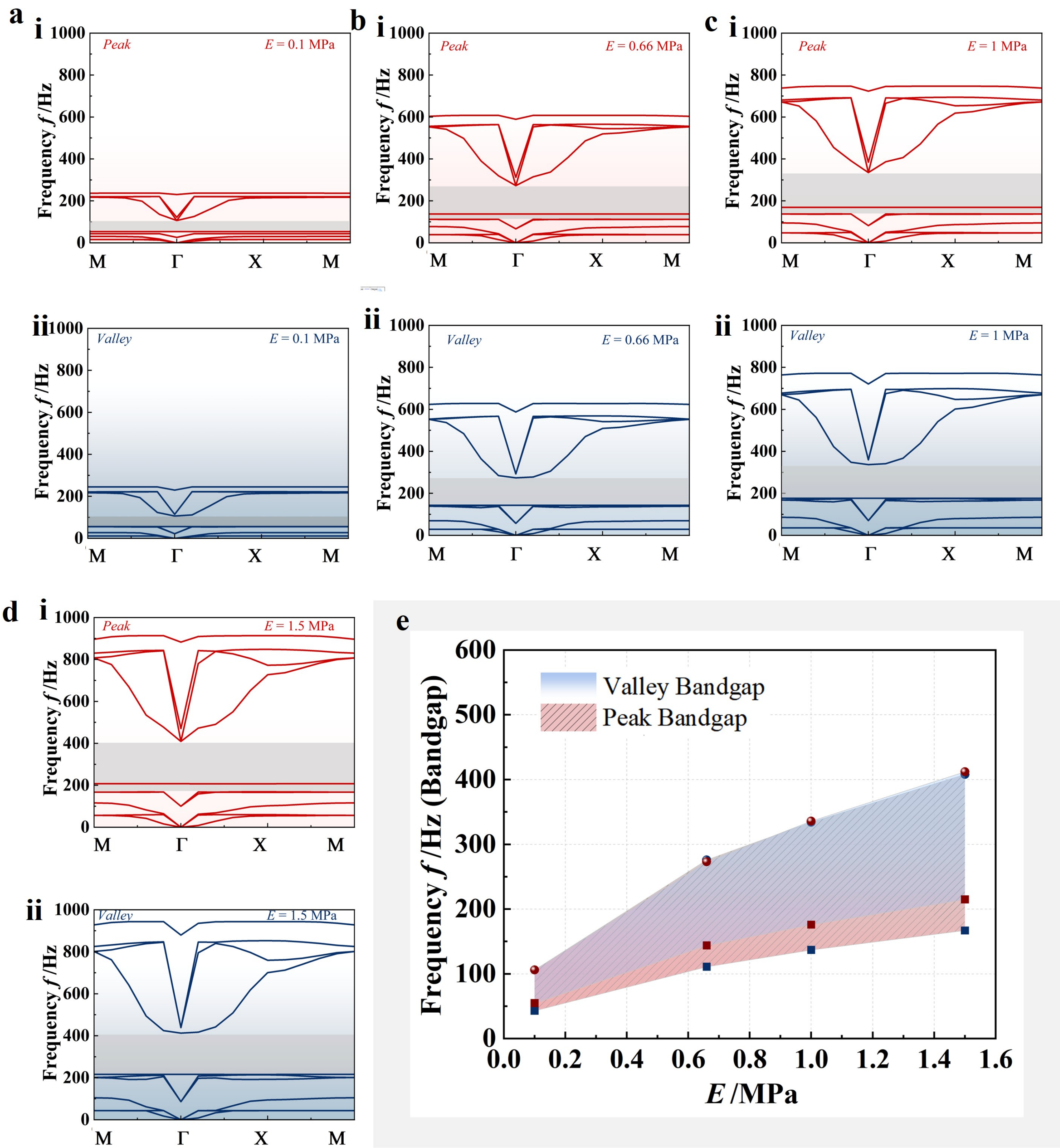

Figure S9: a–d) Calculated dispersion relations for different E, where panels i) and ii) correspond to the peak and valley states, respectively. The gray-shaded regions indicate the complete bandgaps. e) Variation of the bandgap boundaries as a function of E.

Table 2: Effective Bandwidth for Multiple-Defect States

Multi-defect modes	Effective Frequency Range(Hz)	Effective value proportion
SPD	(91, 97), (100.39, 128), (188, 190)	17.8%
DPD	(91,131), (186, 195)	24.5%
LSD	(73, 138), (147, 150), (160, 165), (179, 200)	49.0%
LD	(74,132), (153, 162), (138, 150), (172, 200)	53.5%

S4 Effective Bandwidth for Energy Harvesting in Multiple-Defect States

For the samples exhibiting four programmed multiple-defect states: Single-Point Defect (SPD), Double-Point Defect (DPD), L-Shaped Defect (LSD), and linear defect (LD) modes.
We define an enhancement factor N as follows:

$$N = \frac{A_n}{A_d} \tag{11}$$

where A_d is the received amplitude in the defect state, and A_n is the received amplitude before programming. When $N > 1$, we designate this as the effective frequency region f_{eff}. The efficiency factor η for energy harvesting effective bandwidth is defined as:

$$\eta = \frac{f_{\text{eff}}}{\Delta f} \tag{12}$$

where Δf is the total frequency range under study. We use η to characterize the impact of multiple-defect states on the effective bandwidth for energy harvesting. According to Figure 4, the specific effective frequency range and the proportion of effective frequency are shown in the **Table 2**.

S5 Experimental System

To investigate the acoustic behavior of the ADM, an experimental setup (**Figure S10**) was established which integrated a signal generator (TEKTRONIX, AFG 31000), a power amplifier (WXSA, SA-PA020), and a vibration generator (WXSA, SA-JZ010) as the excitation source, while the vibration response is captured using a laser doppler vibrometer (HUAQIN OPTACOUS, PLV-633) and a data collection card(NI, NI-9234) with high spatial resolution. Stepping movement platforms (FUYU, FLK40), driven by a displacement platform controller (Zolix, ZC300), were employed to adjust the position of the laser vibrometer, enabling vibration signals to be collected at different locations on the fixed specimens. Each unit was topped with laser reflective tape, which served to increase the intensity of the reflected signal and ensure reliable measurements. This setup allows accurate characterization of the dynamic response under various excitation conditions. When demonstrating the elastic waveguide and local damping function through experiments, sinusoidal wave signals with amplitude $V_p = 3\,\text{V}$ and excitation frequency $f_e = 145\,\text{Hz}$ was used.
When conducting experimental research on the transmission coefficients of periodic structures, a swept sinusoidal signal $A_{in}(f_e)$ with a frequency range of f_e = 10–500 Hz and an amplitude of $V_p = 3\,\text{V}$ was applied to the left end of the specimen using the exciter, providing out-of-plane excitation along the Z direction.

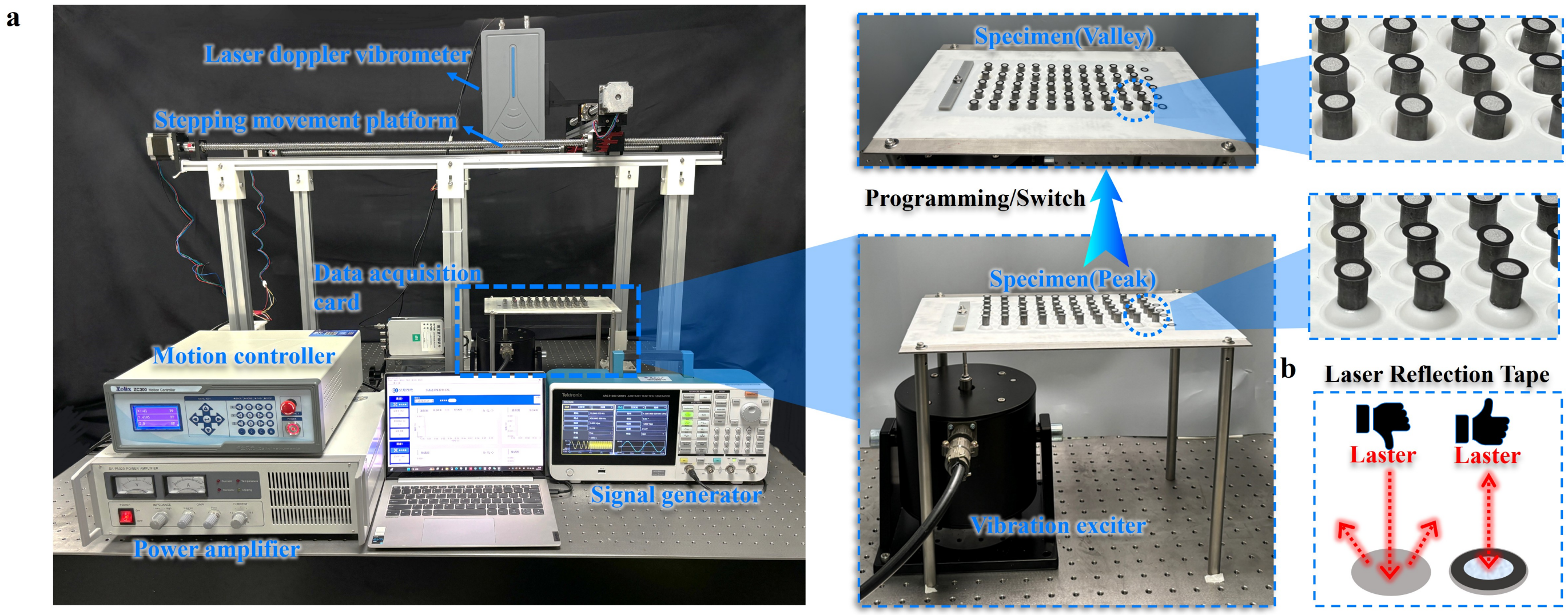

Figure S10: a) The specimens and experimental setup for acoustic characteristic measurements, consisting of a signal generator, a power amplifier, a vibration generator, a laser doppler vibrometer,a data collection card, stepping movement platforms and a displacement platform controller. b) Laser reflective tapes are attached to the top of each unit to improve the quality of the reflected signal.

S6 Mechanic Simulation Parameter Settings

The quasi-static mechanical response of the programmable metamaterial unit cell was analyzed using Abaqus/Standard 2021. The simulation aimed to capture the deformation process, mechanical coupling, and reaction behavior under compression loading. A 3D solid model representing a single metamaterial unit was constructed in Abaqus. The characteristic dimensions were consistent with the experimental prototype, with a typical unit size of 20 mm × 20 mm. The model consisted of three components: a resonant cylinder, an acoustic dome, and a substrate layer.

A reference point (*RP-1*) was defined above the resonant pillar, and its motion was kinematically coupled to the top surface of the pillar in all six degrees of freedom. This setup allowed the extraction of the reaction force–displacement relationship at the coupling point. The interfaces between the cylinder and acoustic dome, and between the acoustic dome and substrate, were defined using tie constraints to ensure fully bonded contact. The bottom surface of the substrate was fixed in all degrees of freedom, while a vertical displacement was applied to the reference point *RP-1* to simulate compression loading. The displacement amplitude was set to 10 mm. The analysis was conducted under quasi-static conditions (Static, General), neglecting inertia effects.

The geometry was discretized using quadratic tetrahedral solid elements C3D10 to accurately capture the local curvature of the dome and the nonlinear deformation near interfaces. While the other parts use the linear hexahedral unit C3D8R mesh. The total reaction force at the reference point *RP-1* was computed to obtain the force–displacement curve, which characterizes the nonlinear stiffness and mechanical programmability of the metamaterial (**Figure S11**a).

S7 Acoustic Simulation Parameter Settings

The acoustic properties, including the dispersion relations and transmission coefficients, were investigated using COMSOL Multiphysics. The simulations were performed within the Solid Mechanics module to capture the elastic wave propagation and resonant behavior of the designed 2D periodic structure.

A ADM lattice with a lattice constant of a = 20 mm was selected as the representative unit cell of the metamaterial (**Figure S11b**). The structure consists solely of solid domains, thus air coupling was neglected.

Periodic boundary conditions were applied along both in-plane directions (x and y axes) using the Floquet–Bloch theorem:

$$\mathbf{u}(\mathbf{r}+\mathbf{a}_i)=\mathbf{u}(\mathbf{r})e^{j\kappa\cdot\mathbf{a}_i} \tag{13}$$

where $\mathbf{a}_i$ are the lattice vectors and κ denotes the Bloch wave vector. Since the first Brillouin zone (BZ) of the ADM is square, the wave vector path was defined along the irreducible Brillouin zone (IBZ), following the sequence M–Γ–X-M. Each segment was sampled at 20 uniformly distributed points to ensure a smooth dispersion curve.

An eigenfrequency study was conducted within the frequency range of 0–500 Hz, and the 10 lowest eigenmodes were extracted at each wave vector. The calculated eigenfrequencies were then combined to construct the band structure, revealing the formation of locally resonant bandgaps (**Figure S11c**).

To validate the dispersion results, a frequency-domain transmission analysis was performed on a finite structure composed of 10×5 cascaded unit cells(**Figure S11d**). A harmonic displacement excitation was applied at the input boundary, providing out-of-plane excitation along the Z direction, while the opposite end was terminated with a perfectly matched layer (PML) to eliminate reflections. The transmission coefficient was defined as the relationship between the incident displacement U_{in} and transmitted displacement U_{out}:

$$T=20log(U_{out}/U_{in}) \tag{14}$$

The 2D model was discretized using Free tetrahedral mesh, with a maximum element size smaller than $\lambda/6$ of the shortest wavelength within the studied frequency range.

In investigating the acoustic properties of the valley state via COMSOL finite element analysis, the deformed geometry obtained from ABAQUS must be imported. This, however, imposes a substantial computational burden due to the added geometric complexity, fine mesh requirements in localized deformations. As the band distribution of the bistable states is predominantly determined by the structure's center of mass (Section S1), a simplified valley-state model is adopted for simulating the periodic structure in the valley configuration.

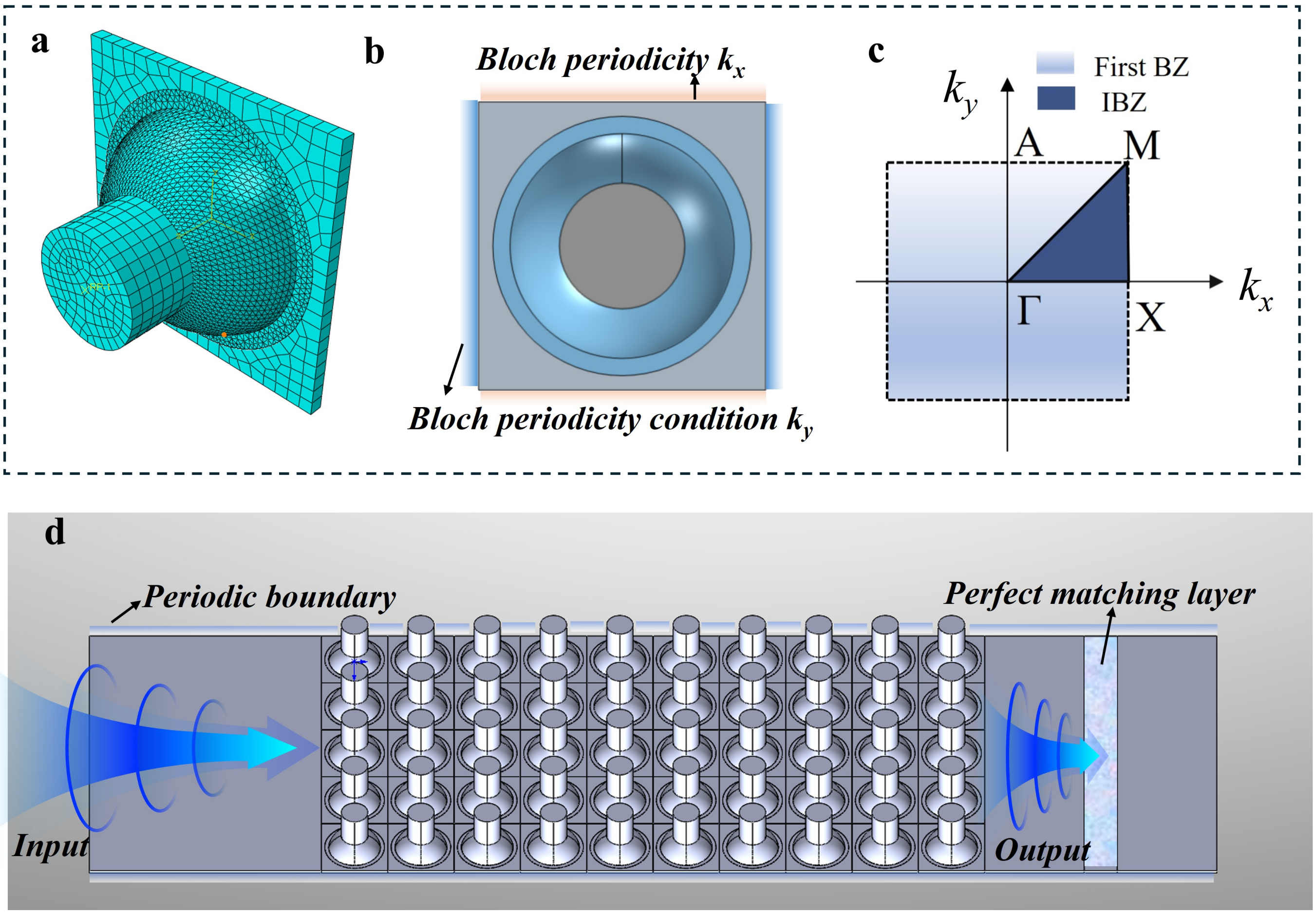

Figure S11: a) ABAQUS finite element simulation mesh generation. b) Schematic of the ADM unit cell and applied periodic boundary conditions. c)Simulated dispersion relation along the high-symmetry path Γ–X–M–Γ, showing the locally resonant bandgap of the ADM. d) Establishment of the periodic structure for calculating the transmission coefficient curve.

S8 AOPP Models

When using the AOPP mechanism for MAI, 3D printing technology can be employed to design models based on the adaptive properties of ADM. This allows the realization of various functions, such as designing binary units with embedded information for data transmission, constructing elastic waveguides for transmission path design, creating localized vibration suppression modules for protecting specific areas, and implementing defect states for energy harvesting. The functional demonstration paths mentioned in this work have all been designed and fabricated, as shown in the **Figure S12**.

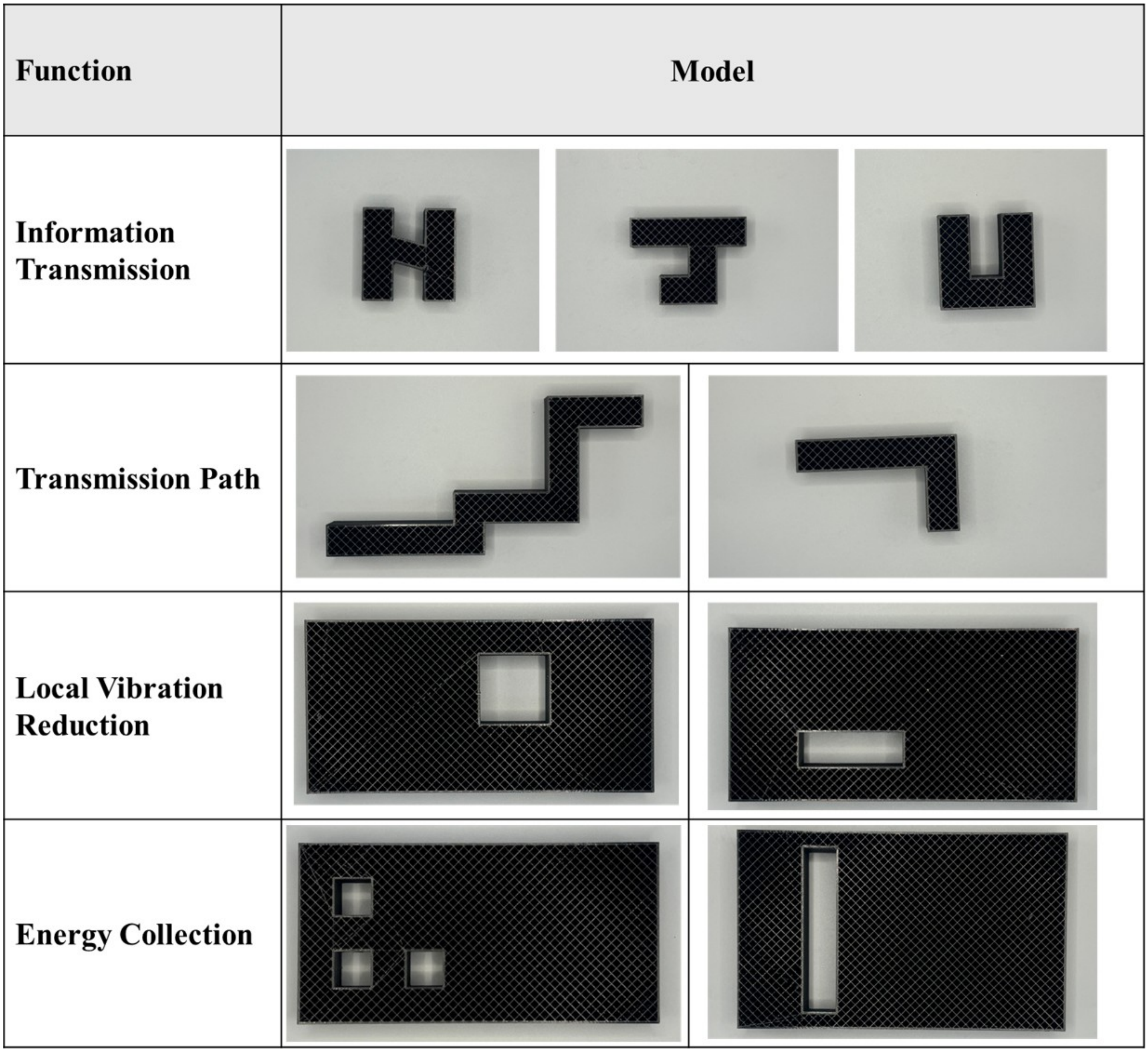

Figure S12: The AOPP 3D printed models corresponding to the acoustic functional demonstrations mentioned in this work.

S9 Error Analysis between Simulation and Experiment

Minor frequency shifts observed between simulated and experimental results are mainly attributed to the following factors:
(1) Adhesive assembly effects

- Variations in bonding layer thickness
- Interfacial friction
- Gaps and microslip at connections

(2) Experimental and modeling approximations:

- Simplified boundary conditions
- Anisotropy of 3D-printed materials
- Vibration transmission through the experimental platform

These factors introduce additional acoustic responses and excite more complex structural vibration modes, leading to deviations from simulations. These factors exert particularly significant influence on the local responses under high-frequency vibration conditions.

References

[1] C. Comi, L. Driemeier, *Latin American Journal of Solids and Structures* **2018**, *15*, 4 e38.

[2] A. S. Fallah, Y. Yang, R. Ward, M. Tootkaboni, R. Brambleby, A. Louhghalam, L. A. Louca, *Wave Motion* **2015**, *58* 101.

[3] M. Kalderon, A. Mantakas, A. Paradeisiotis, I. Antoniadis, E. J. Sapountzakis, *Applied Mathematical Modelling* **2022**, *110* 1.

[4] G. Wang, L.-H. Shao, Y.-Z. Liu, J.-H. Wen, *Chinese Physics* **2006**, *15*, 8 1843.

[5] Y.-F. Wang, Y.-S. Wang, V. Laude, *Physical Review B* **2015**, *92*, 10 104110.

[6] G. Wang, X. Wen, J. Wen, L. Shao, Y. Liu, *Physical Review Letters* **2004**, *93*, 15 154302.

[7] Y.-F. Wang, Y.-S. Wang, L. Wang, *Journal of Physics D: Applied Physics* **2014**, *47*, 1 015502.

[8] Y. Wang, C. Chen, Y. Cheng, *Thin-Walled Structures* **2025**, *216* 113613.

[9] R. Xu, C. Chen, J. Sun, Y. He, X. Li, M.-H. Lu, Y. Chen, *International Journal of Extreme Manufacturing* **2023**, *5* 042013.

[10] C. Zhang, Y. Xu, ..., *Smart Materials and Structures* **2024**, *33*, 9 093001.

[11] M. Taffetani, X. Jiang, D. P. Holmes, D. Vella, *Proceedings of the Royal Society A* **2018**, *474*, 2215 20170910.

[12] E. Ventsel, T. Krauthammer, *Thin plates and shells: Theory, analysis and applications*, Marcel Dekker, New York, NY, USA, **2001**.
